# F2DMAS: a smartphone video-based 3D phenotyping workflow for potted plants in complex backgrounds

**DOI:** 10.3389/fpls.2026.1885579

**Published:** 2026-07-08

**Authors:** Jian Fang, Nengfu Xie, Yane Duan, Jingchao Fan, Xiaoli Wang, Hailong Liu, Huoguo Zheng, Hao Wu, Zhibo Meng, Xin Wang, Rui Man

**Affiliations:** 1 Agricultural Information Institute, Chinese Academy of Agricultural Sciences, Beijing, China; 2 College of Intelligent Science and Engineering, Beijing University of Agriculture, Beijing, China; 3 National Agricultural Science Data Center, Beijing, China; 4 Data Hub, Chinese Agrosystem Long-Term Observation Network, Beijing, China

**Keywords:** 2D gaussian splatting, plant phenotyping, smartphone video, three-dimensional reconstruction, unstructured environment

## Abstract

**Introduction:**

Plant phenotyping requires accurate and repeatable three-dimensional structural information, but practical acquisition conditions in greenhouses, seedling rooms, and indoor pot experiments often include complex backgrounds, handheld motion blur, and thin leaf structures. These factors reduce the robustness of conventional three-dimensional reconstruction methods and limit their use in low-cost and automated phenotyping.

**Methods:**

To address this problem, this paper proposes F2DMAS, an automated three-dimensional plant phenotyping workflow using consumer-grade smartphone videos. The workflow first converts multiview RGB videos into image sequences and removes motion-blurred frames through frequency-domain quality filtering. A frequency–spatial plant segmentation module, termed FSAM3, is then introduced to separate plant structures from complex backgrounds without task-specific annotated training data. The segmented image sequences are further reconstructed using 2D Gaussian Splatting, followed by TSDF-based meshing, scale recovery, and virtual measurement for extracting plant height, canopy width, leaf length, and leaf width.

**Results:**

Experiments were conducted on 15 plant species under two acquisition scenarios. The proposed workflow achieved stable plant reconstruction under non-ideal background conditions, with PSNR, SSIM, and LPIPS values of 31.09, 0.9711, and 0.0365, respectively. Compared with the baseline reconstruction workflow, F2DMAS substantially reduced the processing time for mesh extraction while improving reconstruction quality. The extracted phenotypic traits showed strong agreement with manual measurements, with R² values ranging from 0.90 to 0.99, RMSE values ranging from 0.64 to 1.21 cm, and MAPE values ranging from 4.50% to 9.73%.

**Discussion:**

These results indicate that F2DMAS can provide an end-to-end workflow from smartphone video acquisition and plant segmentation to three-dimensional reconstruction and phenotypic trait extraction. The proposed method offers a practical and deployable solution for greenhouse seedling cultivation, potted plant experiments, and low-cost three-dimensional plant phenotyping.

## 1 Introduction

Plant phenotyping provides fundamental data for crop genetic improvement, cultivation management, and intelligent agricultural decision-making. High-throughput, non-destructive, and high-precision phenotyping methods quantify plant growth processes, canopy structure, stress responses, and varietal differences, thereby supporting crop breeding and production management (Nguyen et al., 2025; Walsh et al., 2024). Traditional phenotypic measurement mainly relies on manual ruler measurement, caliper measurement, and visual recording, which suffer from low efficiency, strong subjectivity, poor repeatability, and possible disturbance to natural plant growth (Zhu et al., 2024). Two-dimensional image analysis improves phenotyping efficiency to some extent, but it remains difficult to accurately characterize three-dimensional structural information such as plant height, canopy width, leaf spatial posture, and canopy volume under canopy self-occlusion, leaf overlap, and complex spatial morphology (Li et al., 2025; Li et al., 2025). Therefore, building a low-cost, repeatable, and automated method for three-dimensional plant phenotyping is an important problem in plant phenotyping research and intelligent agricultural applications (Harandi et al., 2023).

In recent years, three-dimensional sensing and reconstruction techniques, including LiDAR, RGB-D cameras, multi-view photogrammetry, Neural Radiance Fields (NeRF), and Gaussian Splatting, continue to develop and provide multiple technical routes for three-dimensional plant phenotyping (Li et al., 2025; Li et al., 2025; Rincón et al., 2022). However, when these methods move from laboratory algorithm validation to greenhouses, seedling rooms, and ordinary agricultural research scenarios, they still face clear deployment limitations. On the one hand, LiDAR, multi-camera arrays, and some RGB-D devices can acquire high-quality three-dimensional data, but their hardware cost, acquisition requirements, and data processing barriers limit their adoption in small- and medium-scale breeding trials, potted plant studies, and teaching or research scenarios (Jin et al., 2021; Zhu et al., 2021). On the other hand, real or semi-real agricultural environments often contain cluttered backgrounds, illumination changes, similar colors between leaves and backgrounds, blur caused by handheld recording, and variations in plant posture, which easily reduce the stability of three-dimensional reconstruction and subsequent phenotypic measurement (Wang et al., 2026; Yang et al., 2024). In addition, many reconstruction pipelines still require manual background cleaning, model repair, scale calibration, and phenotypic measurement, making it difficult to form a continuous automated process from acquisition and reconstruction to measurement output. For practical applications, the key problem is not only whether a visually high-quality three-dimensional model can be obtained, but also whether analyzable phenotypic data can be collected in batches in a low-cost, minimally invasive, and repeatable manner.

Existing three-dimensional reconstruction methods have their own advantages in plant scenes, but their application conditions and scope remain limited. Structure-from-Motion and Multi-View Stereo (SfM-MVS)-based photogrammetry relies only on RGB images and has the advantages of low cost and flexible acquisition. However, it depends on stable texture and reliable feature matching, and easily produces missing point clouds, background adhesion, and incomplete reconstruction under weakly textured leaves, severely occluded canopies, and complex backgrounds (Li et al., 2025; Wen et al., 2024). NeRF can synthesize novel-view images with strong visual realism, but its implicit representation is sensitive to density thresholds during mesh extraction, and the stability of geometric boundaries and thin sheet-like structures still struggles to meet the needs of phenotypic measurement (Choi et al., n.d; Mildenhall et al., 2022; Zheng et al., 2024). three-dimensional Gaussian Splatting (3DGS) improves rendering efficiency through an explicit Gaussian representation, but its volumetric ellipsoid representation may cause surface thickening, boundary expansion, and normal noise when handling thin-walled organs such as leaves, thereby affecting the measurement of leaf scale and canopy structure (Kerbl et al., 2023). In contrast, two-dimensional Gaussian Splatting (2DGS) uses surface-oriented two-dimensional Gaussian primitives, which are more suitable for representing thin sheet-like structures such as plant leaves and also benefit subsequent explicit mesh extraction (Huang et al., 2024; Yu et al., 2024) However, using 2DGS alone still cannot address input quality fluctuations and background interference in complex agricultural scenes. When raw images contain motion blur, background textures, supports, soil, pots, or other non-target regions, the reconstruction process may allocate substantial representation capacity to irrelevant backgrounds, which increases the difficulty of mesh cleaning and phenotypic measurement.

The development of vision foundation models provides a new way to reduce the cost of plant image segmentation and background removal. Foundation models represented by Grounding DINO, the Segment Anything Model (SAM) series, and related vision-language models show strong zero-shot perception ability and can extract target regions without domain-specific annotated data (Ravi et al., 2024; Singh et al., n.d; Zhu et al., n.d). However, general vision foundation models cannot be directly used as ready-to-use tools in agricultural scenes. Plant images often involve mutual occlusion among leaves, blurred boundaries of small organs, similar colors between targets and backgrounds, continuous viewpoint changes, and unstable illumination. As a result, single-frame segmentation may produce inconsistent multi-view masks, missing targets, or false background segmentation. Meanwhile, blurred frames generated during handheld video acquisition affect SfM feature matching and the stability of subsequent Gaussian reconstruction. Therefore, deployable three-dimensional plant phenotyping requires the systematic integration of image quality filtering, plant segmentation, multi-view reconstruction, meshing, and scale-aware phenotypic measurement, rather than relying on a single algorithmic module.

Recent studies have shown that three-dimensional plant representations can support quantitative phenotyping by capturing geometric traits that are difficult to obtain from two-dimensional images, including plant height, canopy structure, leaf morphology, and organ-level spatial variation (Harandi et al., 2023). Existing image-based plant reconstruction studies have also demonstrated the feasibility of using multi-view images or image sequences for 3D reconstruction and phenotypic trait extraction (Li et al., 2022; Wang et al., 2025). Therefore, F2DMAS is designed as an application-oriented workflow that connects low-cost video acquisition, image preprocessing, 3D reconstruction, explicit meshing, scale recovery, and phenotypic measurement into a continuous pipeline.

To address these problems, this paper proposes F2DMAS, an automated three-dimensional plant phenotyping workflow for unstructured environments. The framework takes multi-view RGB videos captured by consumer-grade smartphones as input and sequentially performs video frame extraction, frequency-domain image quality filtering, plant segmentation driven by vision foundation models, SfM camera pose estimation, 2D Gaussian Splatting-based three-dimensional reconstruction, explicit mesh extraction, scale recovery, and phenotypic trait measurement. Unlike traditional pipelines that depend on dedicated devices or manual post-processing, F2DMAS aims to achieve automated processing from smartphone video acquisition to quantifiable three-dimensional phenotypic output.

The main contributions of this paper are summarized as follows. First, this paper builds an automated three-dimensional plant phenotyping workflow based on consumer-grade smartphone videos, which converts multi-view RGB videos into scale-recovered three-dimensional meshes and phenotypic traits without dedicated imaging hardware or domain-specific annotated data. Second, it integrates fast Fourier transform (FFT)-based frequency-domain frame quality filtering with vision foundation model-based segmentation to form FSAM3, a dual-domain preprocessing module that reduces the effects of handheld motion blur and complex backgrounds on subsequent reconstruction. Third, it combines 2D Gaussian Splatting, Truncated Signed Distance Function (TSDF)-based meshing, and scale recovery to support the automatic extraction of three-dimensional plant phenotypic traits, including plant height, canopy width, leaf length, and leaf width. Fourth, it validates the effectiveness of the workflow on a dataset containing 15 plant species and two acquisition scenarios. The experimental results show that F2DMAS has good application feasibility in terms of reconstruction quality, mesh extraction efficiency, and phenotypic measurement agreement.

## 2 Materials and methods

### 2.1 Overall system architecture

F2DMAS is an automated three-dimensional plant phenotyping workflow designed for unstructured environments. It aims to convert multi-view RGB videos captured by consumer-grade smartphones into scale-recovered three-dimensional models and corresponding measurements for phenotypic analysis. The workflow consists of six stages, including data acquisition, image preprocessing, three-dimensional reconstruction, explicit mesh extraction, scale recovery, and phenotypic trait measurement. Together, these stages form a complete processing pipeline from video acquisition to phenotypic output.

In the data acquisition stage, a consumer-grade smartphone is used to capture multi-view videos around potted plants, and continuous image sequences are extracted from the raw videos. To address motion blur that may occur under handheld recording and non-ideal illumination conditions, the workflow introduces frequency-domain image quality filtering to remove low-quality frames, thereby improving the stability of subsequent feature matching and camera pose estimation. Then, a plant segmentation module is built based on vision foundation models. It obtains plant foreground masks through text prompts and temporal propagation, and separates complex backgrounds, pots, supports, and other non-target regions from multi-view images.

In the three-dimensional reconstruction stage, the preprocessed multi-view images first enter the SfM pipeline to estimate camera intrinsic and extrinsic parameters as well as sparse geometric structures. The workflow then adopts 2D Gaussian Splatting to construct the three-dimensional plant representation, which better matches the surface structure of thin sheet-like plant organs such as leaves. The 2DGS reconstruction process, including SfM sparse point cloud generation, 2DGS reconstruction, outlier removal, surface refining, and high-quality plant mesh generation, is shown in **Figure 2c**. After the optimization of the Gaussian representation, spatial artifact filtering and TSDF mesh extraction are further performed to convert the three-dimensional representation into an explicit mesh model that can be directly measured. Finally, scale recovery is completed using known scale markers in the scene, and basic phenotypic traits, including plant height, canopy width, leaf length, and leaf width, are extracted from the scale-recovered model. The subsequent phenotypic trait computation, including digital-twin scale recovery, plant height, crown diameter, leaf length, leaf width measurement, and validation against ground-truth measurements, is shown in **Figure 2d**. The automatic measurements are compared with manual measurements to evaluate the accuracy and practicality of the workflow for three-dimensional plant phenotyping.

**Figure 1:**
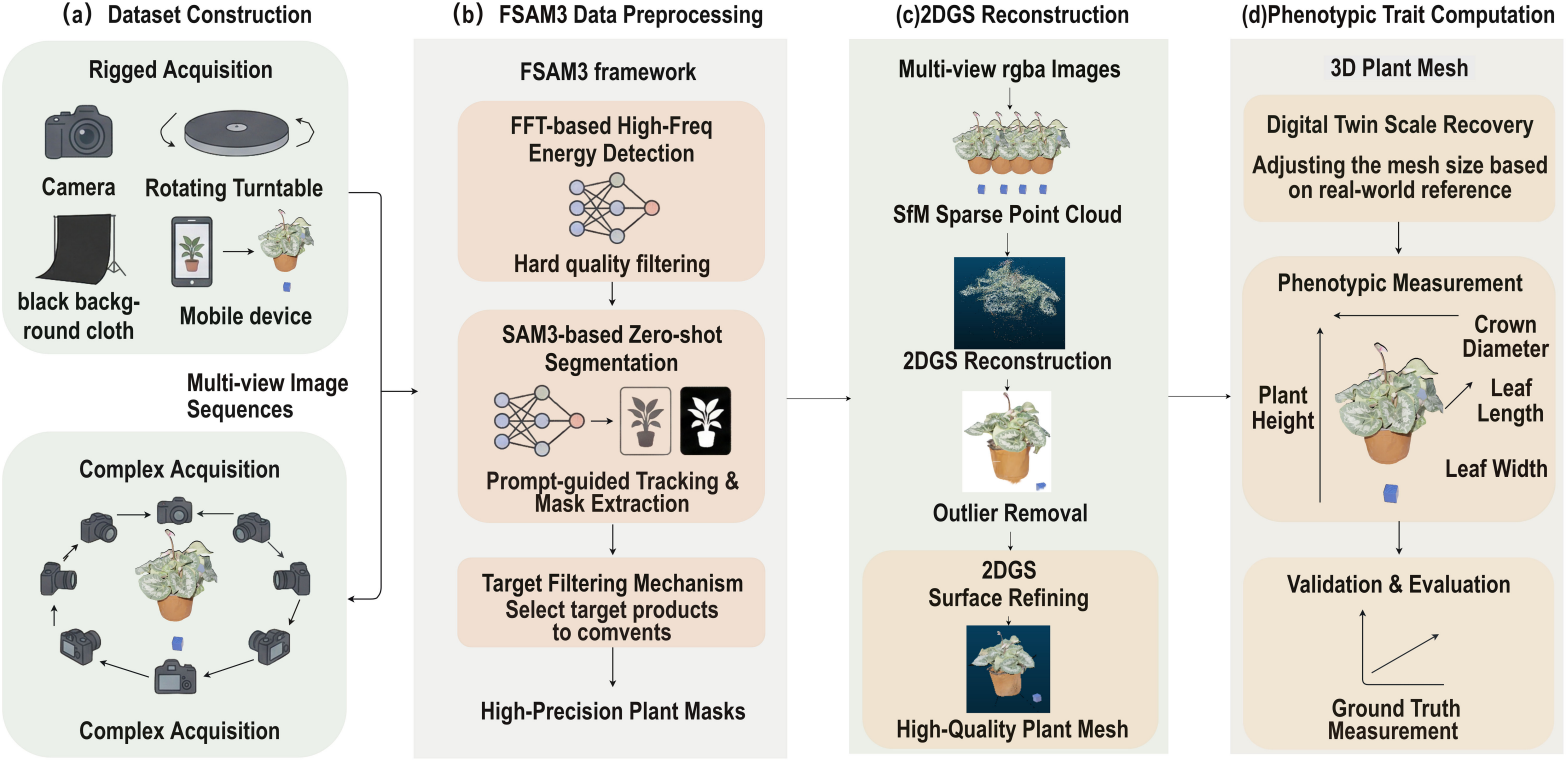
Overall framework of F2DMAS for smartphone video-based 3D plant phenotyping: **(a)** Two Data Collection Methods, **(b)** FSAM3 Framework Workflow, **(c)** 2DGS Reconstruction Process, **(d)** Scale Recovery, Feature Extraction, and Evaluation.

**Figure 1** shows the overall technical pipeline of F2DMAS. Compared with traditional three-dimensional plant phenotyping pipelines that rely on dedicated hardware or substantial manual post-processing, F2DMAS places greater emphasis on low-cost acquisition, automated processing, and continuity in the phenotypic measurement pipeline. The focus of this method is not to propose a single low-level reconstruction algorithm, but to integrate image quality control, plant segmentation, three-dimensional reconstruction, meshing, and phenotypic measurement into a deployable application-oriented system.

### 2.2 Experimental materials and data acquisition

This study constructs a multi-view RGB image dataset for three-dimensional plant phenotyping. Data are acquired in two modes, namely fixed-device-assisted acquisition and handheld acquisition under complex backgrounds. **Table 1** summarizes the plant materials used in this study. The dataset contains 15 plant species with different leaf morphologies, canopy structures, and background interference conditions, and is used to evaluate the applicability of the F2DMAS workflow across diverse plant types and non-ideal acquisition environments. Both acquisition modes use a consumer-grade smartphone for video recording and do not rely on dedicated three-dimensional acquisition devices such as LiDAR, RGB-D cameras, or multi-camera arrays.

**Table 1:** Summary of plant materials.

Sample ID	Plant species	Condition	Scene	Manual
S01	Peace lily	Broad leaves	Fixed	Yes
S02	Strawberry	Low canopy	Fixed	Yes
S03	Strawberry	Overlapping leaves	Fixed	Yes
S04	Kalanchoe	Compact canopy	Fixed	Yes
S05	Kalanchoe	Dense leaves	Complex	Yes
S06	Kalanchoe	Flower-leaf mix	Complex	Yes
S07	Peperomia	Smooth leaves	Fixed	Yes
S08	Peperomia	Thick leaves	Complex	Yes
S09	Peperomia	Dense small leaves	Complex	Yes
S10	Anthurium	Glossy leaves	Complex	Yes
S11	Calathea	Striped leaves	Complex	Yes
S12	Fittonia	Fine texture	Fixed	Yes
S13	Fittonia	Dense texture	Complex	Yes
S14	Chinese evergreen	Broad variegated leaves	Fixed	Yes
S15	Chinese evergreen	Partial occlusion	Complex	Yes
S16	Rubber plant	Large leaves	Fixed	Yes
S17	Rubber plant	Sparse canopy	Fixed	Yes
S18	Cyclamen	Compact canopy	Fixed	Yes
S19	Cyclamen	Flower-leaf mix	Complex	Yes
S20	Cyclamen	Dense occlusion	Complex	Yes

**Figure 2:**
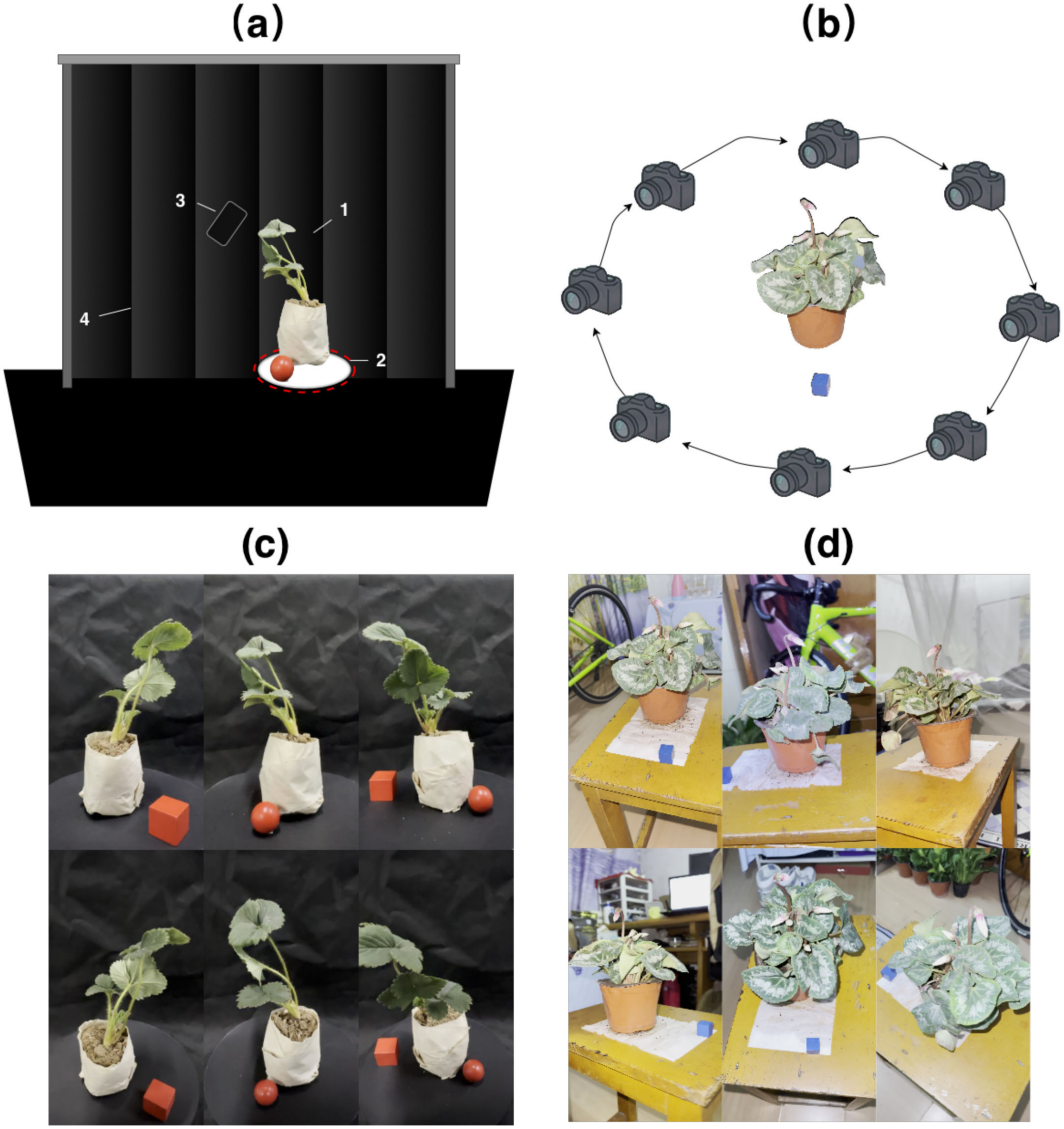
Smartphone-based plant video acquisition protocols under fixed-device-assisted and handheld complex-background conditions **(a)** Fixed-device-assisted acquisition setup, including the target potted plant, motorized turntable, smartphone, black background cloth, and scale marker; **(b)** handheld surrounding acquisition in a complex background, where the target plant is placed in a common indoor or greenhouse-like environment with backgrounds containing tabletops, pots, supports, and other cluttered objects; **(c)** representative image sequence obtained under fixed-device-assisted acquisition; **(d)** representative image sequence obtained under complex-background acquisition. Both acquisition protocols use consumer-grade smartphones for video recording and capture multi-view information from the plant side and canopy top through dual-height surrounding acquisition.

For fixed-device-assisted acquisition, this study builds a low-cost and portable plant video acquisition device, as shown in **Figure 2a**. The device consists of an electric turntable, a black background cloth, a smartphone, and scale markers. During acquisition, the target potted plant is placed at the center of the electric turntable so that the plant rotates slowly with the turntable, while the smartphone remains at a relatively fixed position and continuously records video. Compared with handheld or tripod-based recording around the plant, this setting obtains more stable multi-view image sequences in a smaller space, and is suitable for phenotyping in seedling rooms, laboratory benches, and indoor potted plant experiments. The background cloth reduces interference from irrelevant objects in the environment. During acquisition, wrinkles on the cloth and natural shadows are retained, so the images still contain a certain degree of non-uniform illumination, reflection, and texture disturbance. This design allows the stability of plant segmentation and reconstruction to be evaluated under backgrounds that are not fully controlled.

For complex-background acquisition, plants are placed in ordinary indoor environments or greenhouse-like scenes. The background includes tabletops, supports, pots, walls, floors, and cluttered objects with colors similar to the plant, as shown in **Figure 2b**. The operator holds the smartphone and records video while moving around the target plant, keeping the plant near the image center as much as possible and maintaining a relatively stable field of view for the target plant in most video frames. This acquisition mode does not impose strict constraints on camera height or shooting angle, and is closer to practical scenarios in which smartphones are used for rapid recording of potted plants. It is used to evaluate the adaptability of the workflow to background clutter, handheld motion, viewpoint changes, and illumination fluctuations.

All videos are captured using a consumer-grade iPhone 14 Pro Max smartphone, with a video resolution of 1080 x 1920, a frame rate of 60 FPS, and lens parameters of 13~mm and f/2.2. To improve the structural completeness of three-dimensional reconstruction, each target plant is recorded using a two-height circular acquisition strategy. During acquisition, the plant is first recorded from a lower viewpoint for one or more circular passes to capture side-view information and the outer canopy contour. A second circular pass is then recorded from a higher viewpoint with a certain downward angle to supplement information from the canopy top and overlapping leaf regions. For fixed-device-assisted acquisition, the turntable completes an approximately 360 rotation at each height. For handheld acquisition, the operator completes a continuous circular recording of approximately 360 around the target plant. During acquisition, the plant is kept fully visible in the image as much as possible, and severe motion blur caused by excessively fast movement is avoided.

In practical acquisition, a complete dual-height video sequence for a single plant typically required approximately 1.5-2.0 min under the fixed-device-assisted and handheld complex-background settings. This corresponds to an estimated manual acquisition throughput of about 30–40 plants per hour, excluding sample placement, scale-marker adjustment, and data transfer.

To recover the scale of the three-dimensional model, scale markers or calibration references with known dimensions are placed in the acquisition scene. In fixed-device-assisted acquisition, a black calibration card and a red scale marker are placed near the target plant for subsequent model scaling and conversion to real-world units. In complex-background acquisition, identifiable scale references are placed according to scene conditions, so that the reconstructed model can be converted from relative coordinates to centimeter-scale measurements. After acquisition, the raw videos are extracted into multi-view image sequences and then fed into the subsequent pipeline, including frame quality filtering, plant segmentation, SfM pose estimation, 2DGS reconstruction, and phenotypic measurement.

### 2.3 Frequency-domain frame quality screening

**Figure 3:**
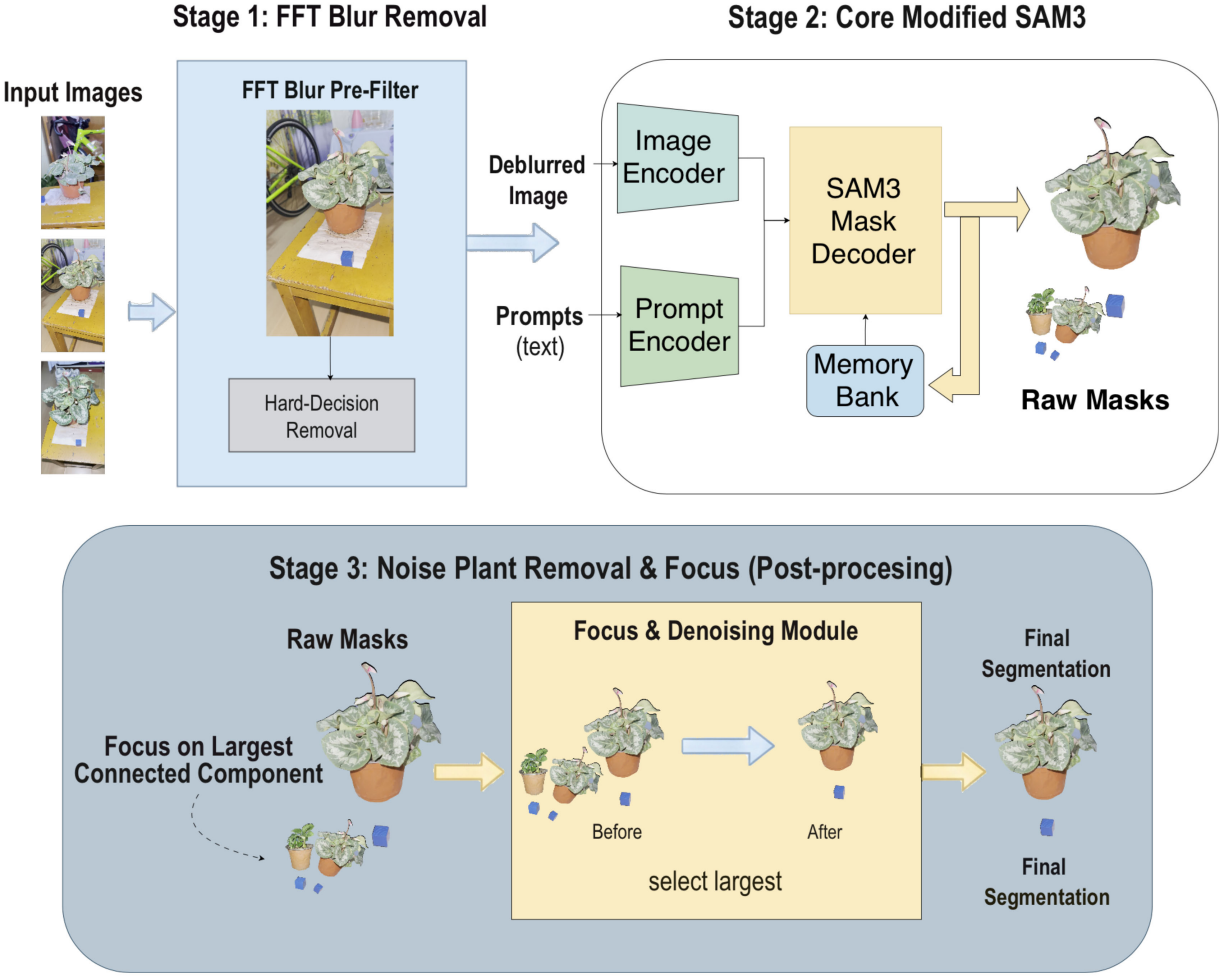
Frequency-spatial preprocessing in F2DMAS for frame filtering and plant foreground.

In video frame-based multi-view plant reconstruction, image sharpness affects feature matching, camera pose estimation, and the stability of subsequent three-dimensional reconstruction (Zhu et al., 2023). In this study, videos are captured using a handheld smartphone or a turntable-assisted setup, and the raw video sequences may contain motion-blurred frames caused by camera shake, slight plant motion, or turntable rotation. If these low-quality images are directly used in SfM and 2DGS reconstruction, they may lead to unstable feature matching, increased camera pose estimation errors, and further degradation in model completeness and phenotypic measurement. Therefore, F2DMAS introduces frequency-domain frame quality filtering during image preprocessing to remove severely blurred frames and improve the reliability of subsequent multi-view geometric computation. As shown in **Figure 3**, the frequency-spatial preprocessing stage consists of frequency-domain frame quality screening and plant foreground extraction. The former removes severely blurred frames according to high-frequency energy, while the latter uses prompt-guided plant segmentation, temporal mask propagation, and morphological post-processing to generate clean plant foreground images for subsequent SfM and 2DGS reconstruction.

Specifically, the input image is first converted into a grayscale image, and a two-dimensional discrete Fourier transform, namely 2D Discrete Fourier Transform, is used to transform the image from the spatial domain to the frequency domain. For a grayscale image *f*(*x*, *y*) with size M x N, its two-dimensional Fourier transform is defined as


$$F\left(u,v\right)=\sum_{x=0}^{M-1}\sum_{y=0}^{N-1}f\left(x,y\right)e^{-j2\pi \left(ux/M+vy/N\right)}$$

The low-frequency components of the spectrum are then shifted to the center, and a logarithmic transformation is applied to the magnitude spectrum to compress the dynamic range:


$$S\left(u,v\right)=\text{log}\left(1+|F_{shift}\left(u,v\right)|\right)$$

Sharp images usually contain rich edge, texture, and detail information, which mainly appears as high-frequency components in the frequency domain. In contrast, motion-blurred or defocused images usually show reduced high-frequency energy. Based on this observation, this paper constructs a low-frequency mask around the spectral center to suppress low-frequency components that mainly reflect global brightness changes, and computes the mean magnitude of the remaining high-frequency region Ω*_high_* as the image sharpness metric:


$$M_{high}=\frac{1}{K}\sum_{\left(u,v\right)\in {\text{Ω}}_{high}}S\left(u,v\right)$$

where *K* denotes the number of pixels in the high-frequency region. For each image frame, if its high-frequency energy metric *M_high_* is lower than a predefined threshold *T*, the frame is identified as a severely blurred frame and removed from the subsequent multi-view image sequence. The filtered image sequence is used for plant segmentation, SfM pose estimation, and 2DGS reconstruction.

Frequency-domain filtering does not provide a comprehensive assessment of all image quality factors. Instead, it serves as a lightweight front-end quality control step to quickly exclude clearly degraded frames that have a strong negative effect on feature matching and three-dimensional reconstruction. This step reduces the interference caused by handheld recording and non-ideal acquisition conditions, and provides a more stable input image sequence for plant segmentation and three-dimensional reconstruction.

### 2.4 Plant segmentation based on vision foundation models

In unstructured or semi-structured plant acquisition scenes, raw images usually contain pots, soil, supports, tabletops, background walls, and other interfering objects with colors similar to the plant. If the complete images are directly fed into the three-dimensional reconstruction pipeline, background regions may participate in camera matching and Gaussian representation optimization, leading to background adhesion, floating artifacts, and redundant structures in the reconstructed model. This also increases the difficulty of subsequent mesh cleaning and phenotypic measurement. Therefore, F2DMAS introduces a plant segmentation module before three-dimensional reconstruction to extract the target plant region from multi-view images and reduce the influence of complex backgrounds on the reconstruction results.

The FSAM3 module constructed in this paper uses the zero-shot segmentation ability of vision foundation models and the temporal propagation mechanism in video sequences to extract the plant body. This design is motivated by recent promptable segmentation foundation models. Segment Anything introduced a general promptable segmentation framework with strong zero-shot transfer ability (Kirillov et al., 2023), while SAM 2 extended promptable segmentation from still images to videos through a streaming-memory mechanism (Ravi et al., 2024). SAM 3 further introduces concept-prompt-based detection, segmentation, and tracking in images and videos, which is consistent with the need to identify plant instances using semantic prompts in multi-view video sequences (Carion et al., 2025). In addition, SEEM provides a promptable segmentation baseline that supports multiple forms of user prompts, including language prompts (Zou et al., 2023).Unlike supervised segmentation methods that depend on a large amount of pixel-level annotated data, this module uses text prompts to guide the model in identifying the target plant region, without retraining the segmentation model for different species or acquisition scenes. In the specific processing procedure, the system inputs a text prompt that describes the target region, such as complete potted-plant foreground excluding only the external background, to guide the model in generating an initial mask of the target plant in the first frame or key frames. The model then uses temporal information and memory propagation in the video sequence to continuously track and update the plant mask across adjacent views, thereby reducing frame-by-frame manual interaction and alleviating inconsistency in single-frame segmentation results.

For an input image *I*, the segmentation module first extracts visual features through an image encoder and converts the text prompt *T* into a semantic prompt vector through a prompt encoder. Combined with temporal memory information *M_memory_*, from historical frames, the mask decoder outputs the predicted plant mask of the current frame:


$$M_{pred}=MaskDecoder\left(ViT\left(I\right),PromptEncoder\left(T\right),M_{memory}\right)$$

Because plant leaf surfaces may contain reflections, occlusions, and local texture loss, the initial segmentation results may include small holes, fragmented boundaries, or locally discontinuous regions. To improve mask completeness, this paper applies morphological closing to the predicted results. Let *B* denote the structuring element. The closing operation is expressed as


$$M_{close}=\left(M_{pred}⊕B\right)⊖B$$

where ⊕ and ⊖ denote dilation and erosion, respectively. This step fills small holes in the plant foreground region and smooths the segmentation boundary to some extent. The processed closed mask is then multiplied with the raw image pixel by pixel to obtain the plant foreground image with the complex background removed:


$$I_{out}=I⊙M_{close}$$

In the practical workflow, *I_out_* is saved as a plant foreground image with an Alpha channel and is used together with the corresponding camera pose information for subsequent 2DGS reconstruction. Through this process, FSAM3 reduces background interference in the raw multi-view images as much as possible, allowing the subsequent three-dimensional representation to focus more on the plant body region.

It should be noted that this paper does not define FSAM3 as a new foundation segmentation model. Instead, it treats FSAM3 as an automatic plant body extraction module within the F2DMAS workflow. This module integrates the zero-shot segmentation ability of vision foundation models, text prompting, temporal mask propagation, and morphological post-processing to meet the foreground extraction requirements of multi-view plant reconstruction. It reduces manual annotation and frame-by-frame interaction, and provides cleaner and more consistent plant image inputs for subsequent camera pose estimation, 2DGS reconstruction, and phenotypic measurement.

After the raw smartphone video is extracted into a multi-view image sequence, severely motion-blurred frames are first removed through frequency-domain high-frequency energy analysis. Then, an initial mask of the plant body is generated using a vision foundation model guided by text prompts, and the target plant is tracked across consecutive views through temporal propagation. Based on this mask, morphological closing is applied to repair local holes and boundary discontinuities. The final plant foreground images with an Alpha channel are used for subsequent SfM pose estimation and 2DGS-based three-dimensional reconstruction.

### 2.5 2DGS-based plant three-dimensional reconstruction

After frequency-domain frame quality filtering and plant segmentation, F2DMAS feeds the processed multi-view plant foreground images into the three-dimensional reconstruction module. This module first uses Structure from Motion (SfM) to estimate camera intrinsic and extrinsic parameters as well as sparse point cloud structures, providing camera poses and initial geometric information for subsequent 2D Gaussian Splatting (2DGS) reconstruction. Compared with direct reconstruction of complete complex scenes, the images preprocessed by FSAM3 reduce interference from pots, supports, background walls, and other non-target regions, which helps focus the reconstruction process on the plant body structure.

Plant leaves, petioles, and canopy boundaries usually exhibit thin sheet-like and surface-dominant geometric characteristics. Traditional 3D Gaussian Splatting (3DGS) represents a scene using three-dimensional volumetric Gaussian primitives. When it handles thin structures such as leaves, it may produce surface thickening, boundary expansion, and adhesion in overlapping regions, thereby affecting subsequent explicit mesh extraction and phenotypic measurement. In contrast, 2DGS describes scene structures using two-dimensional Gaussian primitives oriented along local tangent planes, which is more suitable for representing surface-like organs such as plant leaves. Therefore, this paper adopts 2DGS as the main three-dimensional representation in the F2DMAS workflow to improve the reconstruction stability of thin plant structures and provide depth and normal information for subsequent meshing.

In the 2DGS representation, each Gaussian primitive can be regarded as a local two-dimensional surface patch in three-dimensional space. The primitive consists of parameters such as a three-dimensional center position, local two-dimensional scale, rotation matrix, color, and opacity. The rotation matrix defines the orientation of the local tangent plane, the scale parameters control the spatial extent of the Gaussian primitive on the local surface, and the opacity parameter represents the contribution of the primitive to the final rendered image. Given camera poses and multi-view images, 2DGS optimizes these Gaussian parameters through differentiable rendering so that the rendered results remain consistent with the input images. Its local two-dimensional Gaussian distribution is expressed as:


$$G\left(u\right)=\text{exp}\left(exp\frac{1}{2}u^{T}S^{-2}u\right)$$

where *u* denotes the two-dimensional coordinate on the local tangent plane, and *S* denotes the two-dimensional scale matrix. Unlike volumetric Gaussian representations that perform integration along camera rays, 2DGS focuses more on the geometric relationship between camera rays and local surface patches, and can therefore generate depth maps and normal maps related to plant leaf surfaces.

During training, the system optimizes 2DGS using plant foreground images from different views based on the camera parameters estimated by SfM. The optimization objective mainly includes image reconstruction errors and structural consistency constraints, so that the Gaussian representation can stably reproduce the appearance and geometry of the plant across multiple views. After training, the 2DGS model can not only render plant images from different viewpoints, but also output corresponding depth maps and normal maps. These geometric cues are further used for TSDF mesh fusion, which converts the renderable Gaussian representation into an explicit three-dimensional mesh that can be directly measured.

It should be noted that this paper does not propose 2DGS as a new low-level reconstruction algorithm. Instead, it uses 2DGS as the core three-dimensional representation module in the F2DMAS workflow. The main reason for choosing 2DGS is that its surface-oriented representation is consistent with the structural characteristics of thin sheet-like organs such as plant leaves, while its depth and normal outputs facilitate subsequent mesh extraction and scale-aware phenotypic measurement. By combining FSAM3 preprocessing, SfM camera estimation, 2DGS representation, and explicit meshing, F2DMAS generates plant three-dimensional models suitable for phenotypic analysis from consumer-grade smartphone videos.

**Figure 4:**
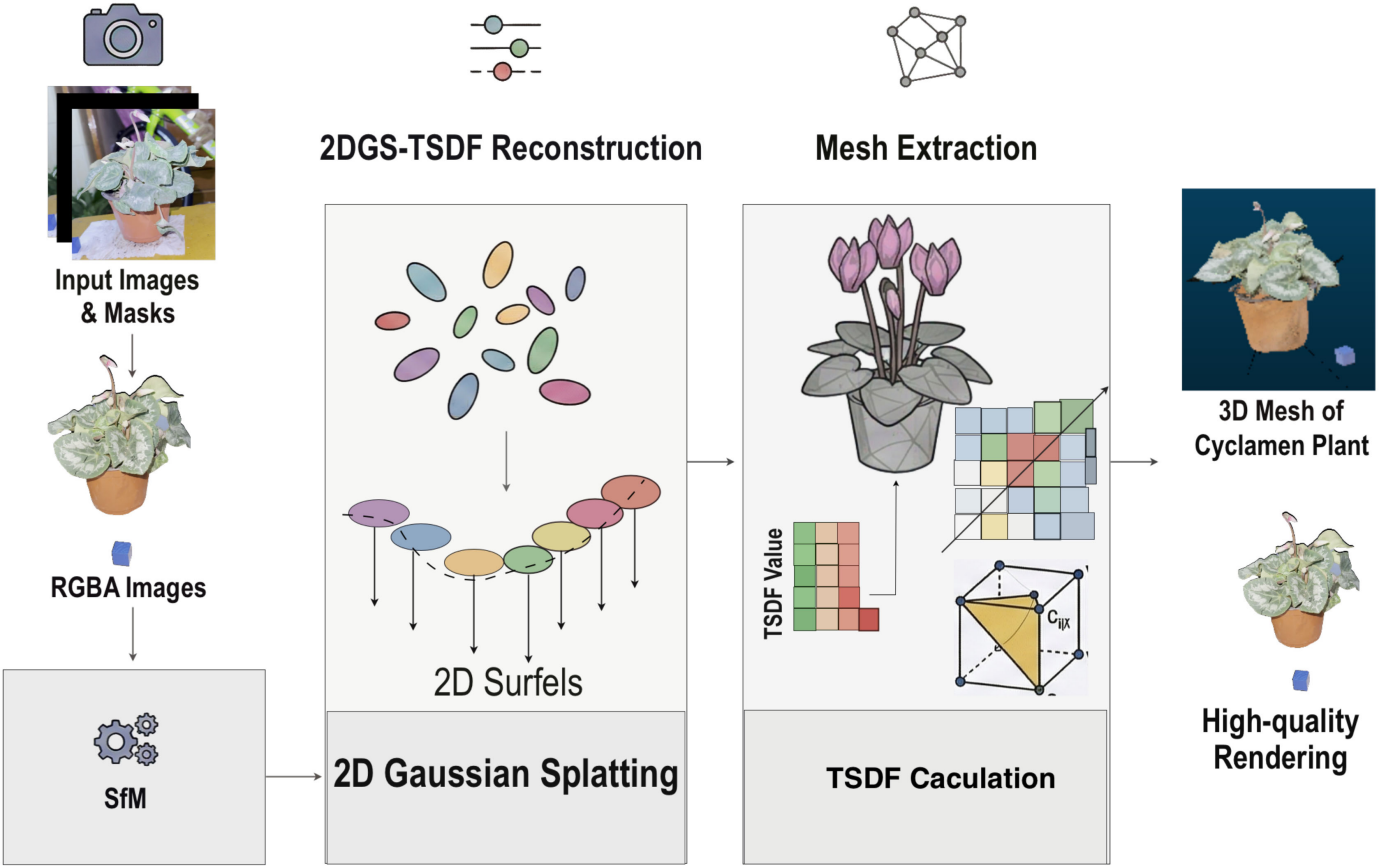
2DGS-based plant 3D reconstruction and geometry map generation pipeline in F2DMAS.

As shown in **Figure 4**, F2DMAS performs 2DGS-based plant 3D reconstruction and geometry map generation after frequency-domain quality filtering and plant instance segmentation. The resulting multi-view foreground images are first fed into the SfM pipeline to estimate camera poses and sparse point cloud structures. The system then uses 2D Gaussian Splatting to construct the 3D plant representation and optimizes the spatial positions, orientations, scales, colors, and opacities of local 2D Gaussian primitives under multi-view image supervision. After training, the model outputs rendered images, depth maps, and normal maps of the plant, providing the geometric basis for subsequent TSDF mesh extraction and phenotypic measurement.

### 2.6 Joint depth-normal explicit meshing

After 2DGS optimization, the system obtains a renderable Gaussian scene representation. This representation can generate multi-view images, depth maps, and normal maps, but plant phenotypic measurement usually requires a measurable explicit three-dimensional geometric model. Therefore, after reconstruction, F2DMAS further performs spatial artifact cleaning and explicit mesh extraction to convert the 2DGS representation into a triangular mesh model for scale recovery and phenotypic measurement.

Under complex backgrounds and multi-view occlusion, a small number of low-contribution or locally abnormal Gaussian primitives may be produced during 2DGS optimization. These artifacts usually appear near leaf occlusion boundaries, regions with insufficient view coverage, or residual background areas, and are observed as dark patches, floating points, or isolated fragments. To reduce their influence on subsequent meshing and phenotypic measurement, this paper adopts a lightweight spatial cleaning strategy before mesh extraction. Specifically, the system extracts the base color components of Gaussian primitives and identifies low-contribution dark artifacts according to perceptual luminance. Gaussian primitives whose luminance is lower than a predefined threshold and whose contribution to the plant body structure is limited are removed during post-processing. This step does not change the basic optimization process of 2DGS, and mainly reduces floating artifacts and local noise before meshing.

The perceptual luminance of a Gaussian primitive is computed from its RGB components as


L=0.299R+0.587G+0.114B

where *R*, *G*, and *B* denote the base color components of the Gaussian primitive, and *L* denotes its perceptual luminance. When *L*< *T_L_*, the primitive is regarded as a potential low-luminance artifact and is filtered out during post-processing. *T_L_* is an empirical threshold that balances the preservation of plant shadow details and the removal of obvious dark noise.

After spatial artifact cleaning, the system uses the depth maps and normal maps rendered by 2DGS for explicit mesh extraction. This paper adopts a Truncated Signed Distance Function (TSDF)-based fusion strategy, which back-projects depth and normal information from multiple training views into a unified three-dimensional space. Specifically, a voxel grid is constructed within the bounding box of the target plant. For each voxel point *x*, its local truncated signed distance value *d_c_*(*x*) is computed using the rendered depth information from different camera views, and the global TSDF value is obtained by weighted averaging:


$$D\left(x\right)=\frac{\sum_{c}w_{c}\left(x\right)d_{c}\left(x\right)}{\sum_{c}w_{c}\left(x\right)}$$

where *c* denotes a camera view, *d_c_*(*x*) denotes the local truncated signed distance value of voxel point *x* with respect to the *c*-th view, and *w_c_*(*x*) denotes the fusion weight corresponding to that view. The weight can be defined according to the consistency between the viewing direction and the surface normal, so as to reduce the influence of unstable depth observations near oblique-view boundary regions on the fusion result.

After the global TSDF field is constructed, the system extracts the isosurface using the Marching Cubes algorithm and generates an explicit triangular mesh of the target plant. Connected component analysis is then used to remove small fragments separated from the main structure, producing the plant mesh model for scale recovery and phenotypic measurement. This mesh model preserves the overall canopy morphology and major leaf structures of the plant body, and provides the geometric basis for virtual measurement of phenotypic traits such as plant height, canopy width, leaf length, and leaf width.

Spatial artifact cleaning and TSDF meshing serve as post-processing steps in the F2DMAS workflow. Their purpose is to convert the renderable 2DGS representation into a measurable explicit geometric model. Topology-aware pruning of Gaussian primitives and boundary correction for thin leaves will be further explored in subsequent methodological studies.

### 2.7 Scale recovery and phenotypic trait measurement

**Figure 5:**
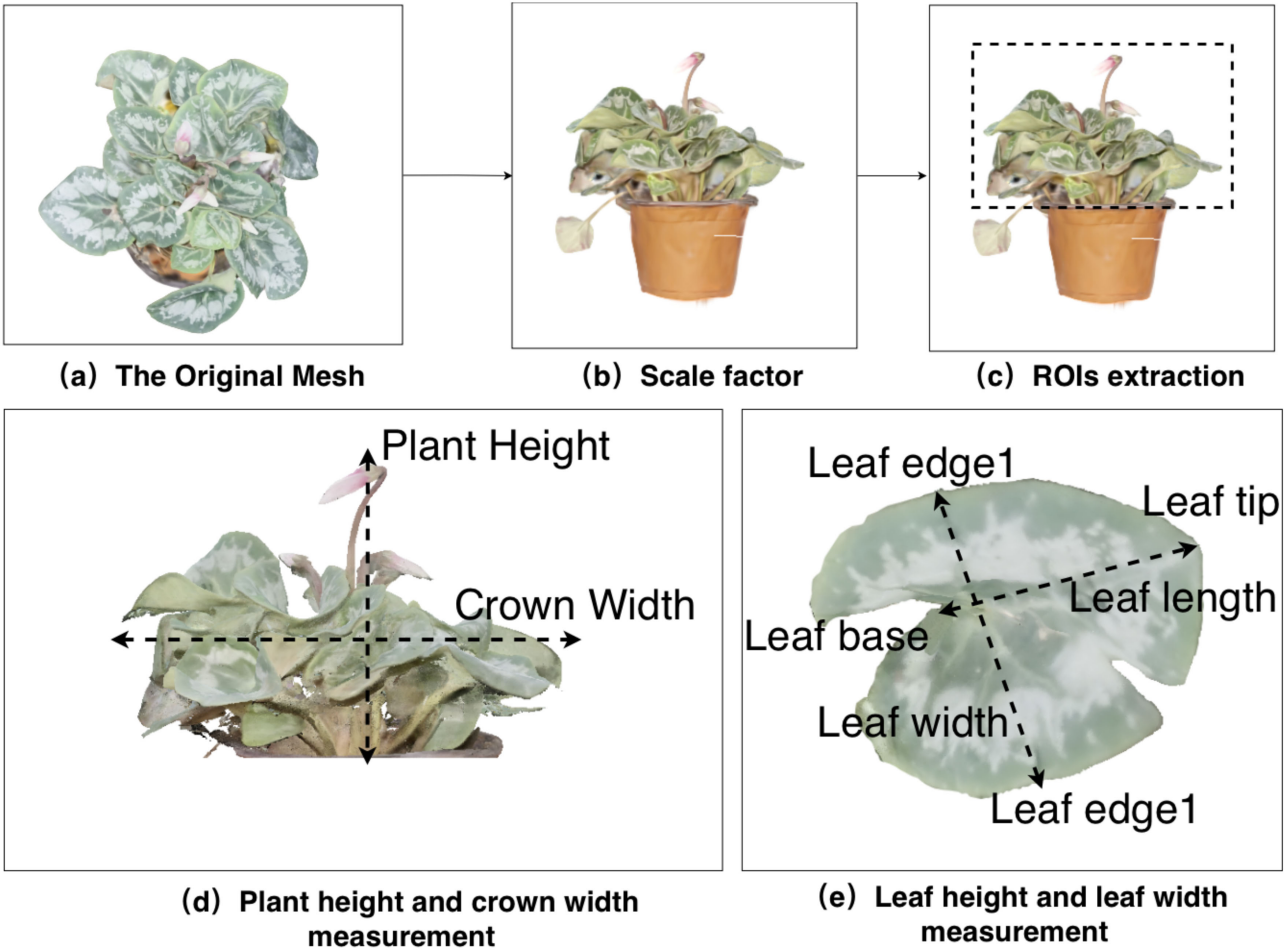
Scale Recovery and Phenotypic Trait Measurement Pipeline for Reconstructed Plant Meshes. **(a)** Plant 3D mesh model before scale recovery; **(b)** calculation of the global scale factor according to the scale marker with a known size in the scene, followed by conversion of the model to the centimeter scale; **(c)** extraction of the plant instance region from the scale-recovered model; **(d)** examples of plant height and canopy width measurement, where plant height denotes the vertical distance from the plant base to the highest point, and canopy width denotes the maximum horizontal extent of the canopy; **(e)** examples of leaf length and leaf width measurement, where leaf length denotes the distance from the leaf base to the leaf tip, and leaf width denotes the maximum width perpendicular to the leaf length direction. The automatic measurements are compared with manual measurements to evaluate the phenotypic measurement accuracy of the F2DMAS workflow.

The three-dimensional models generated by SfM and 2DGS are usually represented in a relative coordinate system, whose spatial units do not directly correspond to real physical scales. To use the reconstructed model for plant phenotypic measurement, the model needs to be converted from a relative scale to real length units. For this purpose, F2DMAS places scale markers with known dimensions near the target plant during data acquisition, and performs scale recovery based on these markers after mesh generation. The scale-recovered three-dimensional plant model is represented in centimeters and can be further used for virtual measurement of phenotypic traits such as plant height, canopy width, leaf length, and leaf width. The overall scale recovery and phenotypic trait measurement pipeline is illustrated in **Figure 5**.

Specifically, let the real length of the scale marker in the acquisition scene be *L_real_*, and let its corresponding virtual length in the reconstructed mesh model be *L_virtual_*. This paper uses point cloud and mesh processing software such as CloudCompare to select the corresponding endpoints of the marker in the reconstructed model, and computes their Euclidean distance as *L_virtual_*. The global scale factor is then calculated from the ratio between the real length and the virtual length:


$$S=\frac{L_{real}}{L_{virtual}}$$

where *S* denotes the coefficient that converts the relative model scale to the real physical scale. After the scale factor is obtained, it is applied to the entire plant mesh model, so that the model coordinates are uniformly converted to the centimeter scale:


$$M_{scaled}=S\cdot M_{original}$$

where *M_original_* denotes the original reconstructed mesh before scale recovery, and *M_scaled_* denotes the scale-recovered three-dimensional plant mesh model. Through this procedure, the three-dimensional model, which originally contains only relative geometric relationships, is mapped to real physical space and provides a scale basis for subsequent phenotypic measurement.

On the scale-recovered model, this paper extracts four basic plant phenotypic traits, namely plant height, canopy width, leaf length, and leaf width. Plant height is defined as the maximum vertical distance of the plant body, which is the distance between the highest point of the plant and the reference plane at the base. Canopy width is defined as the maximum horizontal extension of the plant canopy, which describes the overall lateral growth range of the plant. Leaf length and leaf width are measured within the target leaf region. Leaf length denotes the maximum distance from the leaf base to the leaf tip, while leaf width denotes the maximum leaf width perpendicular to the leaf length direction. For each sample, the virtual measurement procedure follows manual agronomic measurement practices as closely as possible, so that the automatic measurements remain comparable with manual measurements.

To verify the reliability of the phenotypic measurements produced by F2DMAS, this paper compares the virtual phenotypic traits extracted from the scale-recovered models with manual measurements. The manual measurements serve as the reference standard, and the automatic measurements serve as the model outputs. The coefficient of determination *R*^2^, root mean square error (RMSE), mean absolute error (MAE), and mean absolute percentage error (MAPE) are calculated to comprehensively evaluate the measurement accuracy and stability of F2DMAS across different phenotypic traits. This validation process evaluates whether the complete workflow, from smartphone video acquisition, three-dimensional reconstruction, and scale recovery to phenotypic output, can meet the requirements of basic three-dimensional plant phenotyping.

It should be noted that this paper focuses on basic geometric phenotypic measurement for application-oriented scenarios, and therefore selects plant height, canopy width, leaf length, and leaf width as the main validation traits. These traits have clear manual measurement references and can reflect the geometric reliability of the reconstructed model at both the overall canopy scale and the local leaf scale. More complex phenotypic traits, such as leaf area, leaf inclination angle, canopy volume, and plant architecture compactness, can be further extended based on the scale-recovered mesh.

### 2.8 Evaluation metrics

To systematically evaluate the performance of F2DMAS in plant instance segmentation, 3D reconstruction, mesh extraction efficiency, and phenotypic measurement, this study defines metrics from four aspects, namely segmentation accuracy, reconstruction quality, processing efficiency, and phenotypic measurement consistency. Plant instance segmentation is evaluated using F1-score, mean Intersection over Union (mIoU), and HD95, which reflect the completeness of the segmented region, the degree of spatial overlap, and the boundary error. The 3D reconstruction results are evaluated using Peak Signal-to-Noise Ratio (PSNR), Structural Similarity Index Measure (SSIM), and Learned Perceptual Image Patch Similarity (LPIPS), which measure the visual consistency between the reconstructed-view images and the reference-view images. Processing efficiency is characterized by model training time and mesh extraction time. Phenotypic measurement results are evaluated using the coefficient of determination ***R***^2^, Root Mean Square Error (RMSE), Mean Absolute Error (MAE), and Mean Absolute Percentage Error (MAPE), which quantify the consistency between virtual measurements and manual measurements.

In the evaluation of plant instance segmentation, Precision denotes the proportion of pixels predicted as plant foreground that actually belong to the plant region, while Recall denotes the proportion of true plant foreground pixels that are correctly identified. They are defined as follows:


$$P=\frac{TP}{TP+FP}\;R=\frac{TP}{TP+FN}$$

Here, *TP* denotes the number of pixels correctly predicted as plant foreground, *FP* denotes the number of background pixels incorrectly predicted as plant foreground, and *FN* denotes the number of plant foreground pixels that are not correctly identified. F1-score combines Precision and Recall to measure the balance between precision and recall in the segmentation result, and is computed as follows:


$$F1=\frac{2PR}{P+R}$$

mIoU measures the spatial overlap between the predicted region and the manually annotated region, and is defined as follows:


$$mIoU=\frac{1}{k}\sum_{i=1}^{k}\frac{TP_{i}}{TP_{i}+FP_{i}+FN_{i}}$$

Here, *k* denotes the number of classes involved in the evaluation. Since this study mainly focuses on separating the plant instance from the background, mIoU is primarily used to evaluate the segmentation accuracy of the plant foreground region. In addition to region-overlap metrics, HD95 is used to measure the distance error between the predicted boundary and the reference boundary. HD95 is the 95th percentile of the Hausdorff Distance. Compared with the maximum Hausdorff Distance, it is less sensitive to a small number of extreme outliers and is therefore more suitable for reflecting boundary deviations along leaf margins and fine plant structures.

In the evaluation of 3D reconstruction quality, PSNR characterizes the pixel-level error between the reconstructed image and the reference image. The Mean Square Error (MSE) is first computed as follows:


$$MSE=\frac{1}{MN}\sum_{x=1}^{M}\sum_{y=1}^{N}{\left(I\left(x,y\right)-\hat{I}\left(x,y\right)\right)}^{2}$$

Here, *I*(*x*, *y*) denotes the pixel value of the reference image at pixel location (*x*, *y*), $\hat{I}\left(x,y\right)$ denotes the pixel value of the reconstructed image at the corresponding location, and *M* and *N* denote the image height and width, respectively. PSNR is defined as follows:


$$PSNR=10\log_{10}\left(\frac{MAX_{I}^{2}}{MSE}\right)$$

Here, *MAX_I_* denotes the maximum possible pixel value of the image. A higher PSNR indicates a smaller pixel-level error between the reconstructed image and the reference image.

SSIM evaluates image similarity from luminance, contrast, and structure, and is defined as follows:


$$SSIM\left(x,y\right)=\frac{\left(2\mu_{x}\mu_{y}+c_{1})(2\sigma_{xy}+c_{2}\right)}{\left(\mu_{x}^{2}+\mu_{y}^{2}+c_{1})(\sigma_{x}^{2}+\sigma_{y}^{2}+c_{2}\right)}$$

Here, *μ_x_* and *μ_y_* denote the mean luminance values of the two images, $\sigma_{x}^{2}$ and $\sigma_{y}^{2}$ denote their variances, σ*_xy_* denotes their covariance, and *c*_1_ and *c*_2_ are stability constants introduced to avoid division by zero. An SSIM value closer to 1 indicates higher structural similarity between the two images. LPIPS measures perceptual differences between images through distances in a deep feature space, which complements the limitations of pixel-level metrics from a perceptual perspective. A lower LPIPS value indicates that the reconstructed image is closer to the reference image in perceptual feature space.

In the evaluation of processing efficiency, this study records the training time and mesh extraction time of different reconstruction pipelines. Training time reflects the computational cost required to optimize the Gaussian representation from input multi-view images, while mesh extraction time reflects the post-processing cost required to generate an explicit triangular mesh from the reconstructed representation. Together, these two metrics are used to assess the practical efficiency of F2DMAS in batch plant phenotyping scenarios.

In the evaluation of phenotypic measurement, manual measurements are used as reference values, and the virtual measurements extracted by F2DMAS from scale-calibrated 3D meshes are used as predicted values. The coefficient of determination *R*^2^ evaluates the ability of virtual measurements to explain the variation trend of manual measurements, and is defined as follows:


$$R^{2}=1-\frac{\sum_{i=1}^{n}{\left(y_{i}-{\hat{y}}_{i}\right)}^{2}}{\sum_{i=1}^{n}{\left(y_{i}-\overset{ˉ}{y}\right)}^{2}}$$

Here, *y_i_* denotes the manual measurement of the *i*-th sample, ${\hat{y}}_{i}$ denotes the corresponding virtual measurement, ${\hat{y}}_{i}$ denotes the mean of the manual measurements, and *n* denotes the number of samples. An *R*^2^ value closer to 1 indicates higher consistency between virtual measurements and manual measurements.

To further characterize the actual magnitude of measurement errors, this study also computes RMSE, MAE, and MAPE:


$$RMSE=\sqrt{\frac{1}{n}\sum_{i=1}^{n}{\left({\hat{y}}_{i}-y_{i}\right)}^{2}}\;MAE=\frac{1}{n}\sum_{i=1}^{n}|{\hat{y}}_{i}-y_{i}|\;MAPE=\frac{100\%}{n}\sum_{i=1}^{n}|\frac{{\hat{y}}_{i}-y_{i}}{y_{i}}|$$

Here, RMSE is more sensitive to large errors, MAE represents the mean absolute deviation, and MAPE expresses the measurement error in percentage form, which facilitates comparison of measurement stability across phenotypic traits with different scales. These metrics are jointly used to evaluate the accuracy of F2DMAS in basic phenotypic measurement tasks, including plant height, canopy width, leaf length, and leaf width.

### 2.9 Computational environment and implementation details

All experiments were conducted on a workstation running Windows 10 with 128 GB RAM and two NVIDIA RTX A6000 GPUs. The main workflow was implemented in Python 3.11. The 2DGS reconstruction and other GPU-accelerated modules were executed using PyTorch 2.0.0 in a CUDA-enabled environment. This computational environment was used for FSAM3 foreground extraction, 2DGS reconstruction, TSDF-based mesh extraction, scale recovery, and phenotypic trait measurement.

## 3 Result

### 3.1 Data coverage and workflow execution

To evaluate the applicability of F2DMAS across different plant types and acquisition conditions, this study constructs a multi-view plant image dataset using two smartphone-video acquisition protocols, namely fixed-device-assisted acquisition and handheld acquisition in complex backgrounds. The dataset contains 15 plant species and covers diverse conditions in leaf size, canopy compactness, leaf overlap, and background interference. Fixed-device-assisted acquisition provides relatively stable multi-view inputs and is mainly used to evaluate the reconstruction and measurement capability of the workflow under low-cost and relatively controlled conditions. Handheld acquisition in complex backgrounds is closer to unconstrained acquisition scenarios and is used to assess the robustness of F2DMAS under background clutter, view perturbation, and uneven illumination.

For each sample, the original smartphone video is first extracted into a multi-view image sequence. The sequence then undergoes frequency-domain quality filtering, plant instance segmentation, SfM pose estimation, 2DGS-based 3D reconstruction, spatial artifact removal, TSDF mesh extraction, and scale recovery. The workflow finally generates a plant 3D mesh model suitable for phenotypic measurement. In this way, F2DMAS converts RGB videos captured by consumer-grade smartphones into scale-aware 3D models and outputs the corresponding phenotypic trait table, enabling continuous processing from data acquisition to phenotypic output.

**Table 2** summarizes the data coverage and workflow execution under different acquisition scenarios. Overall, F2DMAS completes plant instance segmentation, camera pose estimation, and 3D mesh generation under both fixed-device-assisted acquisition and handheld acquisition in complex backgrounds. In the fixed-device-assisted setting, view changes are relatively smooth and the background is less complex, which usually leads to a higher proportion of valid images and more stable camera registration. In the handheld setting with complex backgrounds, hand motion, background clutter, and illumination changes increase the processing difficulty, but this setting better reflects the applicability of the method under non-ideal acquisition conditions.

**Table 2:** Data coverage and F2DMAS workflow execution across acquisition scenarios.

Scene	Species number	Samples number	Frames	Valid frames	SfM views	Success samples	Success rate
Fixed	8	10	2502	2104	2040	9	90%
Complex	7	10	2500	2113	2089	10	100%

### 3.2 Plant instance segmentation performance in complex backgrounds

Plant instance segmentation is an important preprocessing step in the F2DMAS workflow and directly affects the quality of subsequent processing from raw images to 3D reconstruction. For multi-view plant images captured in complex backgrounds, incomplete target extraction, fragmented boundaries, or incorrectly retained background regions may cause the subsequent 2DGS reconstruction process to allocate part of its representation capacity to non-target regions, such as soil, pots, and supports. This further increases floating artifacts, background adhesion, and the difficulty of mesh post-processing. Based on this consideration, this section evaluates the plant instance segmentation performance of FSAM3 both qualitatively and quantitatively, with SEEM used as a general segmentation baseline.

**Figure 6:**
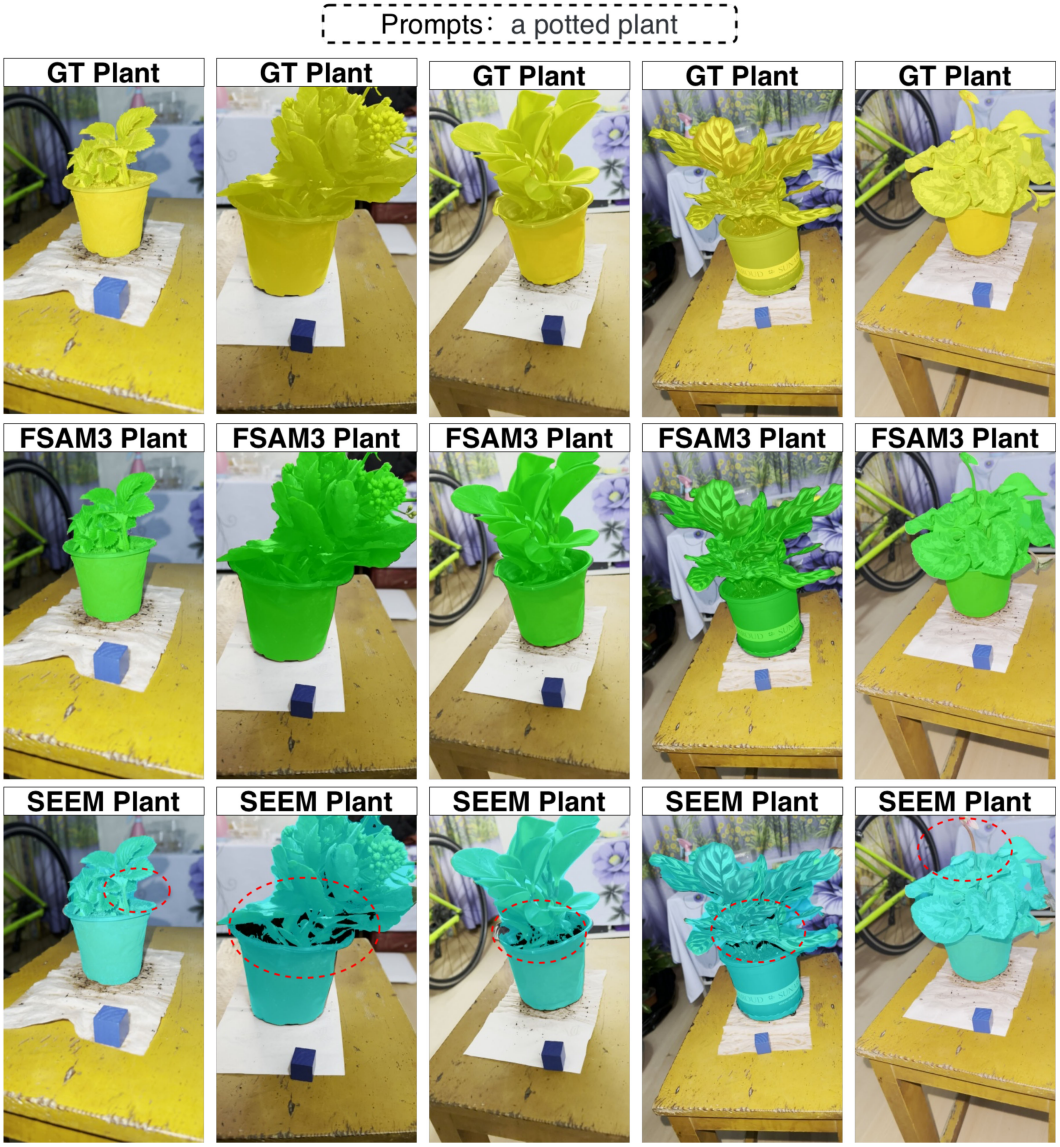
Qualitative comparison of plant instance segmentation results between SEEM and FSAM3 under complex-background conditions.

The qualitative results in **Figure 6** show that FSAM3 extracts the target plant instance more completely and maintains good continuity along leaf margins, canopy contours, and fine structures. For images with leaf overlap, local occlusion, and background color interference, the masks generated by FSAM3 consistently cover the plant foreground region, reducing missing target leaves and boundary discontinuities. In contrast, SEEM shows omissions of fine structures, local leaf loss, and boundary shifts in some samples. When the background texture is complex or the plant region has a color similar to the background, the completeness and continuity of its segmentation results decrease more clearly.

The quantitative results are consistent with these observations. FSAM3 achieves an F1-score of 98.3% and an mIoU of 97.9%, improving over SEEM by 3.2 and 3.8 percentage points, respectively. In terms of boundary error, the HD95 of FSAM3 decreases from 281.9 pixels for SEEM to 41.4 pixels, indicating a substantial reduction in the deviation between the predicted boundary and the reference boundary. The improvements in F1-score and mIoU show that FSAM3 provides better region completeness and overlap in plant foreground recognition, while the lower HD95 indicates more stable localization of boundary regions, such as leaf margins and canopy contours.

The stability of segmentation results directly affects the quality of subsequent 3D reconstruction. A more complete plant foreground mask reduces the probability that background regions enter the 2DGS reconstruction pipeline, allowing the Gaussian representation to focus more on the plant structure and thereby reducing redundant representations and spatial artifacts caused by non-target backgrounds. In complex-background acquisition scenarios, the stable extraction of plant instance boundaries by FSAM3 helps reduce background adhesion and isolated fragments in the resulting mesh, providing a higher-quality input model for scale recovery and phenotypic measurement. Therefore, in the F2DMAS workflow, FSAM3 not only performs plant instance segmentation but also reduces the preprocessing burden of 3D reconstruction and improves the degree of automation of the overall pipeline.

The qualitative comparison in **Figure 6** shows that FSAM3 produces more complete plant masks than SEEM, especially around leaf margins, overlapping regions, and fine plant structures. The quantitative segmentation results are further reported in **Table 3**.

**Table 3:** Performance comparison between FSAM3 and SEEM in plant instance segmentation.

Method	F1-Score	mIoU↑	HD95↓
SEEM	95.1	94.1	281.9
FSAM3	98.3	97.9	41.4
Improvement	+3.2	+3.8	-240.5

### 3.3 Reconstruction quality and processing efficiency with 2DGS

After plant instance segmentation and image quality filtering, F2DMAS estimates camera poses for multi-view images using SfM and then performs 2DGS-based reconstruction. The experimental results show that the image sequences preprocessed by FSAM3 provide stable camera registration results and sparse point clouds. In a representative sample, SfM registers 210 valid camera views and generates a sparse point cloud with 24,226 spatial feature points. This result indicates that frequency-domain frame filtering and plant instance segmentation improve the stability of multi-view reconstruction inputs and reduce the interference of severely blurred frames and complex backgrounds in camera pose estimation.

To evaluate the effect of F2DMAS on 3D plant reconstruction quality and processing efficiency, this study compares it with the standard 2DGS pipeline on the same dataset. Standard 2DGS performs reconstruction directly from raw multi-view images or images in which the foreground is not sufficiently decoupled from the background. In contrast, F2DMAS introduces frequency-domain quality filtering and plant instance segmentation before reconstruction, so that the input content is more concentrated on the target plant region. **Table 4** reports the comparison between the two pipelines in terms of reconstruction quality and processing time.

**Table 4:** Comparison of 3D reconstruction results between F2DMAS and the standard 2DGS.

Metric	PSNR↑	SSIM↑	LPIPS↓	Train time/s ↓	Mesh time(s)
2DGS	29.58	0.9574	0.0487	12913.7	157.9
F2DMAS	31.09	0.9711	0.0365	5044.5	55
Improvement	+1.51	+0.0137	-0.0122	-60.94%	-65.17%

In terms of reconstruction quality, F2DMAS outperforms standard 2DGS on PSNR, SSIM, and LPIPS. Specifically, F2DMAS achieves a PSNR of 31.09 dB, which is 1.51 dB higher than the 29.58 dB of standard 2DGS. SSIM increases from 0.9574 to 0.9711, and LPIPS decreases from 0.0487 to 0.0365. These results indicate that, for plant images captured in complex backgrounds, removing non-target background regions before reconstruction while preserving the plant instance helps improve the consistency between reconstructed views and reference images.

In terms of processing efficiency, F2DMAS reduces both training time and mesh extraction time. The training time of standard 2DGS is 12,913.7 s, whereas that of F2DMAS is 5,044.5 s, which is about 39.1% of the standard 2DGS training time. In the mesh extraction stage, standard 2DGS requires 157.9 s, while F2DMAS requires only 55.0 s. This result shows that non-target regions in complex backgrounds increase the cost of reconstruction optimization and post-processing. By using foreground segmentation to reduce background participation in optimization, F2DMAS allocates more computational resources to the representation of plant structures and thereby improves the overall processing efficiency.

For downstream applications, the role of F2DMAS is reflected not only in improved reconstruction metrics but also in the enhanced usability of the phenotypic measurement workflow. In complex backgrounds, standard 2DGS is easily affected by pots, supports, tabletops, and background textures. Its reconstruction results may contain background residues, floating points, and local noise, which increase the difficulty of mesh cleaning and scale-aware measurement. In comparison, the models generated by F2DMAS contain less background interference and clearer plant structures, providing more stable geometric inputs for subsequent TSDF mesh extraction, scale recovery, and phenotypic measurement of traits such as plant height, canopy width, leaf length, and leaf width.

### 3.4 Comparison of reconstruction quality and processing efficiency

To evaluate the performance of F2DMAS in 3D plant reconstruction, this study compares it with COLMAP, standard 3D Gaussian Splatting (3DGS), and the standard 2DGS pipeline. COLMAP serves as a representative traditional SfM/MVS multi-view geometric reconstruction pipeline. 3DGS represents an explicit neural rendering method based on 3D Gaussian primitives, while standard 2DGS is used to analyze the surface Gaussian reconstruction performance without the complete preprocessing pipeline of F2DMAS. Through these comparisons, the reconstruction quality and processing efficiency of F2DMAS in plant 3D phenotyping scenarios are evaluated from the perspectives of traditional photogrammetry, 3D Gaussian representation, and 2D surface Gaussian representation.

**Table 5** reports the rendering quality and processing time of different reconstruction pipelines on test views. Compared with COLMAP, F2DMAS achieves better results on PSNR, SSIM, and LPIPS. COLMAP obtains a PSNR of 13.63 dB, an SSIM of 0.8745, and an LPIPS of 0.1072, whereas F2DMAS achieves a PSNR of 31.09 dB, an SSIM of 0.9711, and an LPIPS of 0.0365. These results indicate that, under complex plant structures and non-ideal background conditions, it is difficult to obtain stable view reconstruction quality by relying only on traditional sparse and dense geometric reconstruction pipelines.

**Table 5:** Comparison of reconstruction quality and processing efficiency across different reconstruction pipelines.

Method	PSNR (↑)	SSIM (↑)	LPIPS(↓)	Train time(s)	Mesh time(s)
COLMAP	13.63	0.8745	0.1072	599.5	78
3DGS	30.17	0.9587	0.0386	5413.5	642
F2DMAS	31.09	0.9711	0.0365	5044.5	55

Compared with 3DGS, F2DMAS maintains high rendering quality while substantially reducing mesh extraction time. The PSNR, SSIM, and LPIPS of 3DGS are 30.17 dB, 0.9587, and 0.0386, respectively, while F2DMAS achieves 31.09 dB, 0.9711, and 0.0365. In terms of processing efficiency, the mesh extraction time of 3DGS is 642 s, whereas F2DMAS requires only 55 s, corresponding to a reduction of approximately 91.4%. This result shows that, in plant phenotypic measurement tasks, F2DMAS not only provides stable visual reconstruction results but also generates explicit mesh models for subsequent measurement more efficiently.

The comparison with standard 2DGS further shows that the performance gains of F2DMAS mainly come from the optimization of input data by front-end quality filtering and plant instance segmentation. Standard 2DGS obtains a PSNR of 29.58 dB, an SSIM of 0.9574, and an LPIPS of 0.0487, with training time and mesh extraction time of 12,913.7 s and 157.9 s, respectively. F2DMAS increases the PSNR to 31.09 dB and reduces the training time and mesh extraction time to 5,044.5 s and 55 s, respectively. These results indicate that, for plant images captured in complex backgrounds, removing severely blurred frames and separating the plant instance before reconstruction can reduce the interference of background regions in Gaussian representation optimization and mesh extraction, thereby improving the overall efficiency and usability of the workflow.

### 3.5 Workflow ablation study

To analyze the effects of different preprocessing modules in F2DMAS on reconstruction results, this study designs a workflow ablation experiment. The standard 2DGS pipeline using raw frames with background regions and blurred frames retained is used as the Base setting. The FFT-based frequency-domain frame quality filtering module, SAM3-only foreground segmentation, and the complete FSAM3 foreground extraction module are then introduced separately or jointly to form six settings, namely Base, Base+FFT, Base+SAM3, Base+FSAM3, Base+FFT+SAM3, and the full F2DMAS workflow. Here, Base+SAM3 denotes direct foreground extraction using SAM3 masks without the additional FSAM3 post-processing steps, whereas Base+FSAM3 denotes the use of the complete FSAM3 module, including SAM3-based mask generation and subsequent foreground refinement. By comparing PSNR, SSIM, LPIPS, training time, and mesh extraction time under different settings, this experiment evaluates the roles of image quality filtering and plant foreground segmentation in the 3D reconstruction pipeline.

**Table 6** reports the ablation results. Compared with Base, adding FFT-based frequency-domain frame quality filtering increases PSNR from 29.58 dB to 29.80 dB, increases SSIM from 0.9574 to 0.9623, and decreases LPIPS from 0.0487 to 0.0453. The training time and mesh extraction time also decrease from 12,913.7 s and 157.9 s to 12,510.3 s and 144.3 s, respectively. These results indicate that removing severely blurred frames improves the quality of input images to some extent and reduces the influence of low-quality views on feature matching and Gaussian optimization. However, the improvement brought by frame quality filtering alone remains limited. Because background regions are still retained in the reconstruction images and can still participate in 2DGS optimization and TSDF meshing.

**Table 6:** Ablation results of FFT-based frame filtering and FSAM3 foreground extraction using the same 2DGS reconstruction backend.

Method	FFT	SAM3	FSAM3	2DGS	PSNR↑	SSIM↑	LPIPS↓	Training time/s↓	Mesh time/s↓
Base	×	×	×	✓	29.58	0.9574	0.0487	12913.7	157.9
Base+FFT	✓	×	×	✓	29.80	0.9623	0.0453	12510.3	144.3
Base+SAM3	×	✓	×	✓	30.14	0.9656	0.0426	7826.4	92.8
Base+FSAM3	×	×	✓	✓	30.50	0.9687	0.0397	6541.3	73.6
Base+FFT+SAM3	✓	✓	×	✓	30.37	0.9672	0.0411	7314.8	84.5
Base+FFT+FSAM3(F2DMAS)	✓	×	✓	✓	31.09	0.9711	0.0365	5044.5	55.0

After introducing SAM3-only foreground segmentation, the improvements become more evident. Base+SAM3 increases PSNR to 30.14 dB and SSIM to 0.9656, while reducing LPIPS to 0.0426. The training time decreases from 12,913.7 s in Base to 7,826.4 s, and the mesh extraction time decreases from 157.9 s to 92.8 s. These results show that, for plant images captured in complex backgrounds, background regions increase the computational cost of Gaussian representation optimization and mesh extraction. By separating the plant instance from the background, SAM3-only foreground extraction allows the reconstruction process to focus more on the target plant region, thereby improving both reconstruction quality and processing efficiency. However, SAM3-only masks may still suffer from incomplete plant segmentation, plant-background adhesion, noisy connected regions, and residual artifacts under complex backgrounds. After replacing SAM3-only masks with the complete FSAM3 foreground extraction module, Base+FSAM3 further improves PSNR to 30.50 dB and SSIM to 0.9687, reduces LPIPS to 0.0397, and decreases the training time and mesh extraction time to 6,541.3 s and 73.6 s, respectively. This comparison indicates that the performance gain is not simply caused by using SAM3 as the segmentation backbone, but also comes from the additional FSAM3 foreground refinement steps.

Under FFT-filtered input conditions, Base+FFT+SAM3 achieves a PSNR of 30.37 dB, an SSIM of 0.9672, and an LPIPS of 0.0411, with training and mesh extraction times of 7,314.8 s and 84.5 s, respectively. The full F2DMAS workflow, which combines FFT-based frame quality filtering and FSAM3 foreground extraction, achieves the best results across all metrics. Its PSNR reaches 31.09 dB, SSIM reaches 0.9711, and LPIPS decreases to 0.0365. The training time and mesh extraction time decrease to 5,044.5 s and 55.0 s, respectively. Compared with Base, the full workflow reduces training time by 60.94% and mesh extraction time by 65.17%. These results indicate that FFT-based frame quality filtering and FSAM3-based foreground extraction are complementary. The former mainly reduces the influence of blurred frames on multi-view geometric estimation and reconstruction optimization, while the latter mainly reduces background interference, noisy mask regions, and plant-background adhesion in Gaussian representation and TSDF meshing. When combined, these two modules improve both reconstruction stability and processing efficiency of F2DMAS under non-ideal acquisition conditions. Importantly, Base+FSAM3 uses the same raw-frame condition as Base, with blurred frames retained, but still outperforms Base and Base+SAM3. This comparison confirms that the improvement of F2DMAS is not solely due to FFT-based blur-frame removal or the direct use of SAM3, but results from the combined contribution of FSAM3 foreground refinement and FFT-based frame quality filtering.

### 3.6 Mesh quality analysis in overlapping leaf regions

In addition to rendering metrics and processing time, 3D plant phenotyping also requires attention to whether the reconstructed model preserves geometric structures that are useful for measurement. For potted plants and seedling canopies, leaf occlusion and overlap are important factors that affect 3D phenotypic measurement. If the reconstructed mesh shows adhesion, blurred boundaries, or local surface swelling in overlapping leaf regions, the extraction of phenotypic traits such as leaf length, leaf width, canopy width, and canopy structure is directly affected. Therefore, this study further conducts a qualitative comparison of plant meshes generated by different methods, with emphasis on geometric recovery in overlapping leaf regions.

**Figure 7:**
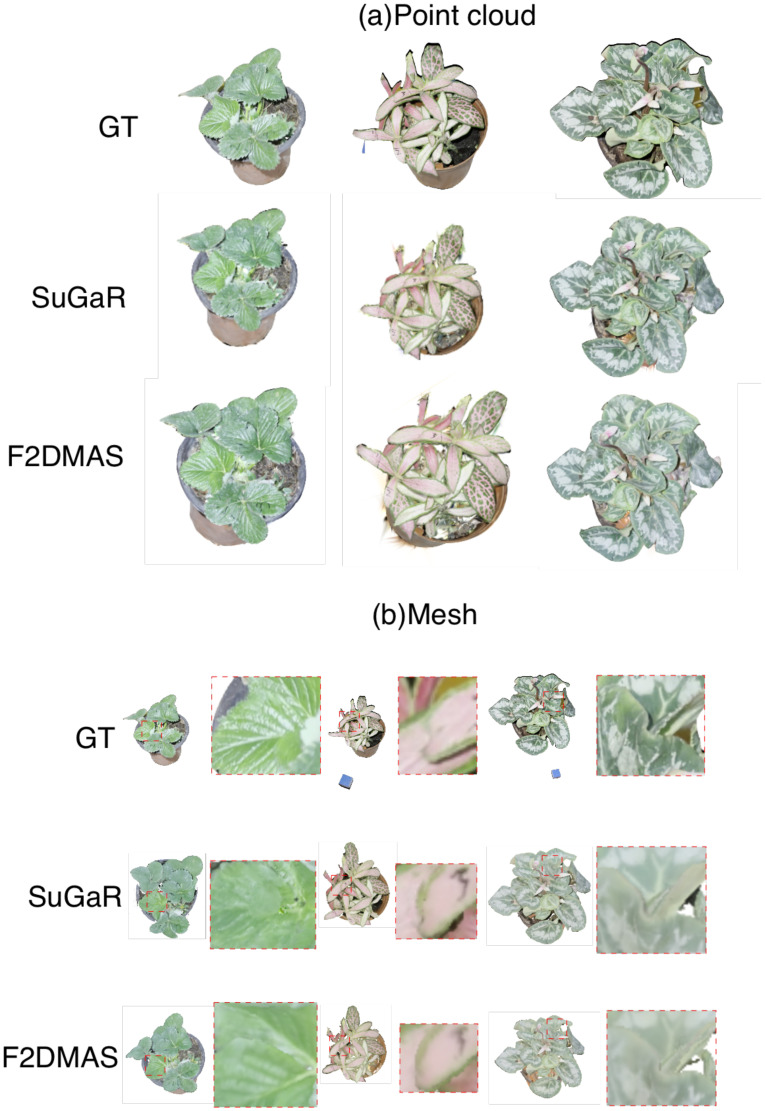
Comparison of Plant Mesh Reconstruction Results in Overlapping Leaf Regions across Different Methods **(a)** Qualitative Comparison of Gaussian Point Clouds Across Original Images, SuGaR, and F2DMAS. **(b)** Comparison of Surface Meshes for Phenotypic Analysis.

As shown in **Figure 7**, the target plant in the original image contains clear leaf overlap and canopy occlusion. The mesh generated by SuGaR recovers the overall morphology of the plant instance, but boundary blurring and local adhesion tend to appear in leaf intersection regions, and the spatial separation between adjacent leaves is not sufficiently clear. These problems affect subsequent leaf-level phenotypic measurement. In particular, when identifying leaf boundaries or extracting local leaf width, adhesive regions may lead to overestimated measurements or reduced measurement stability.

In contrast, the mesh generated by F2DMAS is more concentrated on the plant instance region, with fewer background residues and isolated fragments. In overlapping leaf regions, F2DMAS preserves the boundary relationships between adjacent leaves more clearly and reduces abnormal adhesion between leaves, making the canopy structure and major leaf contours more suitable for subsequent virtual measurement. This result indicates that the combination of FSAM3-based foreground decoupling and 2DGS surface representation not only improves rendering quality but also enhances the usability of explicit meshes for plant phenotypic measurement.

It should be noted that F2DMAS does not fully solve fine-grained leaf geometry recovery. In very thin leaf margins, oblique-view regions, and severely occluded areas, the mesh may still show local boundary shifts or slight surface swelling. This observation is consistent with the relatively higher error and positive bias observed in subsequent leaf width measurement. Therefore, this study positions F2DMAS as a deployable workflow for 3D plant phenotyping. Boundary correction and topology optimization for thin leaf structures remain important directions for future methodological research.

### 3.7 Phenotypic measurement accuracy

**Figure 8:**
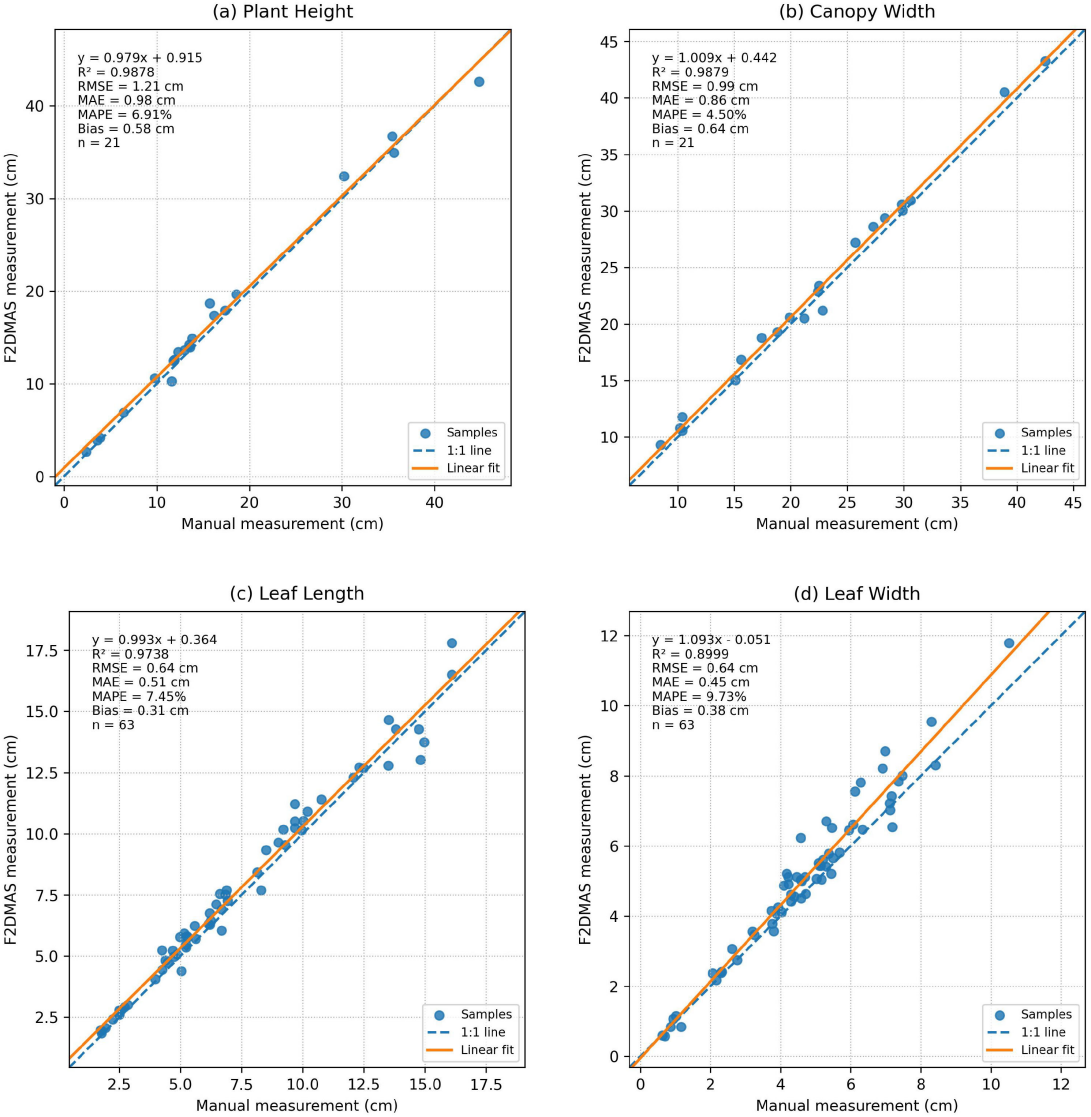
Comparative results of phenotypic analysis.

To verify whether the 3D meshes generated by F2DMAS support plant phenotypic analysis, this study extracts four basic phenotypic traits from scale-recovered models, namely plant height, canopy width, leaf length, and leaf width, and compares them with manual measurements. **Figure 8** shows the regression relationships between virtual measurements and manual measurements for different phenotypic traits, and **Table 7** reports the quantitative comparison between F2DMAS-based phenotypic measurements and manual measurements. The results show that plant height, canopy width, and leaf length achieve high agreement with manual measurements, with *R*^2^ values of 0.99, 0.99, and 0.97, respectively. In contrast, leaf width shows a lower *R*^2^ of 0.90, indicating that fine-grained local traits are more sensitive to reconstruction boundary errors and mesh surface quality.

**Table 7:** Comparison between F2DMAS-based phenotypic measurements and manual measurements.

Phenotypic traits	N	Manual mean/cm	R²↑	RMSE/cm ↓	MAE/cm ↓	MAPE/% ↓	Bias/cm→ 0
Plant height	21	16.26	0.99	1.21	0.98	6.91	0.58
Canopy width	21	22.30	0.99	0.99	0.86	4.50	0.64
Leaf length	63	7.25	0.97	0.64	0.51	7.45	0.31
Leaf width	63	4.68	0.90	0.64	0.45	9.73	0.38

The measurement stability differs across phenotypic traits. For canopy-scale traits, plant height achieves an RMSE of 1.21 cm, an MAE of 0.98 cm, a MAPE of 6.91%, and a positive bias of 0.58 cm. Canopy width achieves an RMSE of 0.99 cm, an MAE of 0.86 cm, a MAPE of 4.50%, and a positive bias of 0.64 cm. These results indicate that F2DMAS can stably recover the overall vertical and lateral extent of the plant canopy. Plant height and canopy width mainly depend on the overall plant contour and the outer canopy boundary, and are therefore less affected by local errors along leaf margins.

Leaf-level local traits are more difficult to measure than canopy-scale traits. Leaf length achieves an *R*^2^ of 0.97, an RMSE of 0.64 cm, an MAE of 0.51 cm, a MAPE of 7.45%, and a positive bias of 0.31 cm, showing relatively stable agreement with manual measurements. Leaf width obtains the lowest *R*^2^ among the four traits, with an *R*^2^ of 0.90, an RMSE of 0.64 cm, an MAE of 0.45 cm, a MAPE of 9.73%, and a positive bias of 0.38 cm. This result suggests that leaf width is more affected by depth instability near leaf margins, surface expansion under oblique views, and boundary dilation during TSDF meshing.

These results show that F2DMAS supports the extraction of canopy-scale phenotypic traits, such as plant height and canopy width, and provides feasible measurements of leaf-scale traits, such as leaf length and leaf width. However, compared with plant height, canopy width, and leaf length, leaf width shows lower agreement and a larger relative error, indicating that fine-grained leaf boundary reconstruction remains the main limitation of the current workflow. Therefore, this study positions F2DMAS as an application-oriented workflow for 3D plant phenotyping, with emphasis on verifying the feasibility of the complete process from smartphone video input to basic phenotypic output. Future work should further improve thin-leaf boundary correction and reduce local phenotypic measurement errors.

## 4 Discussion

### 4.1 Application significance of F2DMAS for low-cost 3D plant phenotyping

F2DMAS proposed in this study is not an isolated algorithm designed only to optimize reconstruction metrics. Instead, it is a complete workflow that covers smartphone video acquisition, 3D model generation, and phenotypic trait output. In greenhouse seedling cultivation, potted-plant experiments, and indoor plant phenotyping studies, the acquisition of 3D structural data is often limited by sensing equipment, environmental conditions, and post-processing cost. LiDAR, multi-camera systems, and some RGB-D devices can provide high-quality 3D data, but their hardware cost, acquisition configuration, and data processing requirements remain relatively high. As a result, they are difficult to deploy widely in small- and medium-scale breeding trials, teaching and research activities, and routine plant monitoring. In contrast, F2DMAS only relies on consumer-grade smartphones to capture multi-view RGB videos and does not require dedicated 3D sensors or complex acquisition platforms, thereby lowering the practical barrier to 3D plant phenotyping from the data acquisition stage.

This positioning is consistent with recent reviews showing that image-based plant phenotyping is moving toward low-cost sensing, automated analysis, promptable/foundation models, and deployable workflows, while still facing challenges in cost, annotation demand, model generalization, and field robustness (Jin et al., 2025; Wang et al., 2025).

From the perspective of the application workflow, the role of F2DMAS is not limited to generating plant 3D models, but also lies in converting the models into quantifiable phenotypic data. Traditional 3D reconstruction pipelines often take model visualization as the main output, while subsequent steps still require manual background cleaning, model repair, scale setting, and trait measurement. F2DMAS connects video frame extraction, image quality filtering, plant instance segmentation, camera pose estimation, 2DGS reconstruction, mesh extraction, scale recovery, and phenotypic measurement into a continuous process from acquisition to reconstruction, measurement, and result output. The experimental results show that this workflow outputs basic phenotypic traits such as plant height, canopy width, leaf length, and leaf width, and maintains high consistency with manual measurements. This indicates that the goal of F2DMAS is not simply to obtain reconstructed images with higher PSNR, but to provide an executable 3D data production workflow for agricultural research and plant phenotypic analysis.

For practical users, this workflow is easy to understand and execute. In a typical greenhouse seedling or potted-plant experiment, researchers can use a smartphone to capture a dual-height surrounding video of a single plant for 1–2 min and upload the video to a computing server. The system then automatically performs frame filtering, plant segmentation, 3D reconstruction, mesh generation, and phenotypic measurement. The final outputs include a scale-aware 3D mesh model, visualization results, and a phenotypic trait table. This mode is suitable for small- and medium-scale breeding trials, controlled-environment plant experiments, teaching demonstrations, and the accumulation of 3D plant phenotypic data.

### 4.2 Role of frequency- and spatial-domain preprocessing in deployability

Under non-ideal acquisition conditions, input image quality and plant instance segmentation directly affect subsequent 3D reconstruction. Smartphone video acquisition lowers the hardware barrier, but it also introduces hand motion, motion blur, exposure variation, and background clutter. If low-quality images and complex backgrounds are directly fed into SfM or 2DGS, they may lead to unstable camera pose estimation, background participation in Gaussian optimization, and floating points or background adhesion in the resulting mesh. Therefore, front-end preprocessing is not a simple image enhancement step, but a key component that determines whether the workflow can run automatically and stably.

The frequency-domain frame quality filtering module in F2DMAS is mainly used to remove frames with severe motion blur. Although this step is lightweight, it has a clear role in smartphone-based acquisition scenarios. It reduces the interference of low-quality views in feature matching and camera registration, and provides a more stable input sequence for subsequent SfM and 2DGS. The ablation study also shows that adding FFT-based filtering alone improves both reconstruction quality and processing time to some extent. This indicates that, in a workflow designed for practical deployment, even a relatively simple quality control step can contribute positively to overall stability.

Compared with frequency-domain filtering, plant instance segmentation has a more substantial effect on workflow efficiency and result quality. In complex backgrounds, non-target regions such as pots, soil, supports, tabletops, and walls occupy Gaussian representation capacity and increase the difficulty of mesh post-processing. FSAM3 combines the zero-shot segmentation capability of a visual foundation model with temporal propagation in video sequences, producing relatively stable plant foreground masks without relying on manual annotations. Compared with single-frame segmentation, temporal propagation helps maintain target-region consistency across multi-view sequences and reduces reconstruction instability caused by abrupt mask changes between adjacent views. The segmentation results not only outperform SEEM in F1-score, mIoU, and HD95, but more importantly provide cleaner plant-instance inputs for subsequent 3D reconstruction, reducing the probability that background regions enter the 2DGS optimization and TSDF meshing processes.

Recent studies have also shown the value of combining vision foundation models with neural 3D representations for plant phenotyping. For example, IPENS lifts SAM2-generated masks into NeRF space for target point-cloud extraction (Song et al., 2025). In contrast, F2DMAS uses FSAM3 as a preprocessing module before 2DGS optimization, so that background regions are suppressed before Gaussian representation capacity is allocated.

Therefore, the role of FSAM3 in F2DMAS should not be understood only as improving segmentation accuracy. It should be viewed as a front-end input purification mechanism for deployable 3D reconstruction. This mechanism reduces the cost of manual background cleaning and makes multi-view plant images more suitable for automated reconstruction. This is also one of the main advantages of F2DMAS over directly using standard 2DGS.

### 4.3 Applicability of 2DGS to plant surface reconstruction

The requirements of 3D plant phenotypic measurement differ from those of conventional novel view synthesis. For phenotypic analysis, the model needs not only good visual realism, but also geometric structures that can support measurement, such as canopy outlines, leaf boundaries, spatial separation between leaves, and the overall plant scale. Plant leaves usually have thin-sheet structures and surface-dominant geometry, which are closer to 2D surfaces than to volumetric structures. Therefore, whether a 3D representation can effectively describe thin leaf structures directly affects subsequent mesh extraction and phenotypic measurement.

3DGS represents a scene using 3D ellipsoidal Gaussian primitives and provides high rendering efficiency. However, surface thickening and adhesion between adjacent structures tend to occur near leaf boundaries, occluded regions, and thin-sheet structures. For tasks mainly focused on image rendering, such local geometric errors may not be obvious. In phenotypic measurement tasks such as leaf length, leaf width, and canopy width estimation, however, boundary expansion and leaf adhesion directly affect the measurement results. In contrast, 2DGS uses 2D Gaussian primitives oriented to local surfaces, which better match the geometry of thin-sheet organs such as leaves. It also outputs depth maps and normal maps, providing more suitable geometric information for subsequent TSDF meshing.

Recent Gaussian-Splatting-based plant studies further confirm that Gaussian representations are becoming useful tools for plant visualization and phenotyping. PlantGaussian explores 3DGS for cross-time and cross-scene plant visualization, Cotton3DGaussians uses multiview 3DGS for cotton boll mapping and plant architecture analysis, and LCR-GS converts greenhouse 3DGS scenes into plant-level units for muskmelon trait extraction (Jiang et al., 2025; Lin and Lin, 2026; Shen et al., 2025). Compared with these 3DGS-centered studies, F2DMAS emphasizes surface-oriented 2DGS and TSDF meshing for thin, leaf-like structures, which is more directly aligned with leaf-scale phenotypic measurement.

The mesh visualization results in this study also support this analysis. In overlapping leaf regions, SuGaR or reconstructions based on volumetric Gaussians tend to show boundary blurring and local adhesion, while the mesh generated by F2DMAS preserves the separation between adjacent leaves more clearly. This difference has a direct impact on plant phenotypic measurement. Whether leaves remain relatively independent in the mesh affects the virtual extraction of traits such as leaf length, leaf width, and canopy structure. Therefore, F2DMAS selects 2DGS not simply to pursue better rendering metrics, but to obtain a 3D representation that is more suitable for explicit meshing and phenotypic measurement.

At the same time, 2DGS does not fully solve all problems in plant geometry reconstruction. For fine leaf margins, heavily occluded regions, and leaf boundaries under oblique views, the current mesh may still show local expansion or boundary shifts. This problem is evident in leaf width measurement, where the virtual measurements show the lowest R² among the four traits, together with a MAPE of 9.73% and a positive bias of 0.38 cm. This observation indicates that F2DMAS can already support canopy-scale and partial leaf-scale phenotypic measurement, but there remains room for further optimization in high-precision local leaf geometry recovery.

### 4.4 Deployment pathway for agricultural research scenarios

The potential application scenarios of F2DMAS mainly include greenhouse seedling cultivation, potted-plant experiments, controlled-environment phenotyping studies, and teaching and research platforms. Unlike large-scale field high-throughput phenotyping platforms, these scenarios usually have moderate sample sizes and limited acquisition space, but are more sensitive to equipment cost, operational complexity, and data processing efficiency. Researchers need to obtain 3D models and basic phenotypic traits of individual plants at relatively low cost, while avoiding complex hardware setup and extensive manual post-processing.

In practice, F2DMAS can adopt a cloud-edge collaborative deployment mode. The user side mainly performs low-cost data acquisition, using a smartphone to record multi-view videos around the target plant. The computing side performs image filtering, plant segmentation, camera pose estimation, 2DGS reconstruction, mesh extraction, and phenotypic measurement. This mode avoids the need for high-performance GPUs on the user side while retaining the flexibility of smartphone acquisition. For breeding researchers, the final system output is not only a single 3D visualization model, but also a phenotypic trait table suitable for statistical analysis, including basic geometric traits such as plant height, canopy width, leaf length, and leaf width.

This deployment mode helps transform a complex computer vision pipeline into a data acquisition tool that is easier for agricultural users to operate. In small-scale breeding trials, teaching experiments, and dynamic observations of plant growth, researchers can periodically collect videos of the same batch of potted plants and use F2DMAS to generate comparable 3D phenotypic records. Although the current study does not yet cover real field scenarios or continuous full-life-cycle monitoring, the combination of smartphone acquisition and automated processing provides a feasible pathway for low-cost 3D plant phenotypic data acquisition.

### 4.5 Limitations and future work

Although F2DMAS achieves good reconstruction quality, mesh extraction efficiency, and phenotypic measurement consistency on the dataset used in this study, several limitations remain. First, the current data mainly come from potted plants, fixed-device-assisted acquisition, and complex indoor-background acquisition, and do not yet cover real field scenarios. Field environments involve stronger natural illumination variation, wind-induced plant motion, complex soil backgrounds, and multi-plant occlusion, which may have greater effects on image segmentation, camera pose estimation, and 3D reconstruction. Therefore, the stability of the workflow still needs to be further verified in greenhouse production environments and real field conditions.

This limitation is also consistent with recent 3DGS-based plant phenotyping studies, where robustness can decrease under denser canopy structures, support-structure entanglement, changing illumination, or task-specific residual background noise (Lin and Lin, 2026).

Second, the current dataset does not cover the complete growth period of plants, and mainly verifies 3D reconstruction and basic phenotypic measurement under single-acquisition conditions. For plant growth analysis, stress response monitoring, and breeding selection, the consistency of 3D phenotypes across time series is also important. Future studies can perform continuous acquisition at different growth stages of the same plants to evaluate the repeatability and sensitivity of F2DMAS in temporal phenotypic analysis.

Third, the current phenotypic traits mainly focus on basic geometric traits such as plant height, canopy width, leaf length, and leaf width. These traits have clear manual measurement references and are suitable for validating the basic measurement capability of the workflow, but they are not sufficient to cover all needs in plant phenotyping research. In the future, scale-aware meshes can be further used to extract 3D phenotypic traits such as leaf area, canopy volume, leaf inclination angle, plant compactness, and stem morphology, and to explore their relationships with biomass, growth status, and cultivar differences.

Fourth, leaf-width measurement remains more challenging than plant height, canopy width, and leaf length because it is highly sensitive to local boundary geometry. To examine whether this limitation was mainly introduced by mesh extraction, we additionally compared TSDF-mesh-based leaf-width measurement with Gaussian-point-cloud-based measurement on a representative thin-leaf sample. The Gaussian point-cloud representation reduced the measurement error compared with the mesh-based result, suggesting that TSDF fusion and mesh extraction can contribute to boundary smoothing or slight dilation near thin leaf margins. However, residual error remained after using the Gaussian point-cloud representation. This observation indicates that the relatively higher error in leaf-width measurement is not caused by meshing alone, but also reflects upstream uncertainty, including small segmentation boundary shifts, 2DGS depth instability under oblique views, sparse boundary points, and local ambiguity around thin leaf edges. Therefore, future work should further investigate plant-aware Gaussian optimization, topology-aware pruning, and edge-aware meshing to improve fine-grained leaf phenotypic measurement.

Fifth, FSAM3 still relies on text prompts and the generalization capability of visual foundation models. When the background is highly complex, the plant and background have very similar colors, or the plant is severely occluded, a single prompt may be insufficient to obtain stable masks. Future work can further investigate prompt ensembling, lightweight domain adaptation, and few-shot fine-tuning strategies to improve the stability of plant instance segmentation across more agricultural scenarios.

Finally, F2DMAS still requires relatively high computational resources, and the inference of visual foundation models and 2DGS optimization are more suitable for cloud servers or workstations. Therefore, future work can optimize the overall efficiency through model lightweighting, parallel task scheduling, and batch processing system design, making the workflow more suitable for medium- and large-scale plant phenotypic data production. Meanwhile, to address boundary expansion, positive bias, and relatively high MAPE in leaf width measurement, future research further explores plant-aware Gaussian optimization, topology-aware pruning, and edge-aware meshing to improve the accuracy of fine-grained leaf phenotypic measurement.

## 5 Conclusion

This study proposes F2DMAS, a smartphone-based 3D plant phenotyping workflow for unstructured acquisition environments. The workflow takes multi-view RGB videos captured by consumer-grade smartphones as input and sequentially performs frame quality filtering, plant instance segmentation, SfM camera pose estimation, 2D Gaussian Splatting-based 3D reconstruction, spatial artifact removal, TSDF mesh extraction, scale recovery, and phenotypic trait measurement. It enables automated processing from smartphone video acquisition to the output of scale-aware 3D models and phenotypic data. Compared with traditional pipelines that rely on dedicated 3D sensors or extensive manual post-processing, F2DMAS emphasizes low-cost acquisition, automated processing, and deployability for phenotypic measurement.

The experimental results show that F2DMAS provides stable plant instance segmentation and 3D reconstruction results for plant images captured in complex backgrounds. The front-end FSAM3 segmentation module achieves an F1-score of 98.3% and an mIoU of 97.9%, with a markedly lower HD95 than SEEM, indicating that it extracts the plant instance region more completely. In terms of reconstruction quality and processing efficiency, compared with standard 2DGS, F2DMAS improves PSNR and SSIM by 5.10% and 1.43%, respectively, and reduces LPIPS by 25.05%. Meanwhile, it reduces training time and mesh extraction time by 60.94% and 65.17%, respectively. Compared with 3DGS, F2DMAS reduces mesh extraction time by 91.4%, showing higher processing efficiency for explicit mesh generation and batch phenotyping scenarios.

For phenotypic measurement, F2DMAS extracts basic traits such as plant height, canopy width, leaf length, and leaf width from scale-recovered 3D meshes. Plant height, canopy width, and leaf length achieve high agreement with manual measurements, with R² values of 0.99, 0.99, and 0.97, respectively. Leaf width shows a lower R² of 0.90 and the highest MAPE of 9.73%, indicating that fine-grained leaf-width measurement remains more sensitive to local boundary reconstruction and meshing errors. Overall, the workflow reliably supports basic 3D plant phenotypic analysis, especially for canopy-scale traits, while thin-leaf boundary correction remains an important direction for further improvement.

Overall, F2DMAS provides a low-cost and automated solution for 3D plant phenotyping in greenhouse seedling cultivation, potted-plant experiments, controlled-environment phenotyping studies, and teaching and research scenarios. Future work further extends the workflow to real field scenarios and full-growth-period validation, and improves fine-grained leaf geometry reconstruction and phenotypic measurement accuracy through plant-aware Gaussian optimization, topology-aware pruning, and edge-aware meshing.

## Data Availability

The raw data supporting the conclusions of this article will be made available by the authors, without undue reservation.
